# Small Ruminant Farms as Managed Ecosystems: A Conceptual Framework for Components and Interactions, and the Role of Health Management

**DOI:** 10.3390/ani16193067

**Published:** 2026-09-29

**Authors:** Eleni I. Katsarou, Dimitrios C. Chatzopoulos, Maria V. Bourganou, Natalia G. C. Vasileiou, Ilektra A. Fragkou, Vasia S. Mavrogianni, George C. Fthenakis

**Affiliations:** 1 Veterinary Faculty, University of Thessaly, 43100 Karditsa, Greece; elekatsarou@vet.uth.gr (E.I.K.);; 2 Faculty of Public and One Health, University of Thessaly, 43100 Karditsa, Greece; 3 Faculty of Animal Science, University of Thessaly, 41110 Larissa, Greece

**Keywords:** abiotic, biotic, ecosystem, environmental compartment, farm ecosystem, flow, goat, health management, infection, interaction, pathogen, sheep

## Abstract

Livestock farms are a distinctive type of agricultural ecosystem wherein farmed animals interact continuously with other living organisms and with the physical environment of the farm under the influence of human management. The objectives of this paper are the proposal of a framework in which small ruminant farms are considered as managed ecosystems, the identification, definition and characterization of the principal biotic components and abiotic environmental compartments, the examination of interactions occurring within and between these components and the description of the role of health management in modifying interactions within these systems. Within the proposed approach, the ecosystem of small ruminant farms can be considered to be characterized by five principal elements: components and compartments, interactions and flows and their directionality, inputs and outputs crossing the physical or functional boundaries of the system, and human management and feedback. Small ruminant farms can function as networks of ecosystem interactions, in which complex interactions occur among and between their biotic components and abiotic environmental compartments. In brief, the biotic components of the farm ecosystem include the livestock (sheep, goats), other domestic or synanthropic animals on farms, wildlife near farms, people working on farms or visiting, vegetation and other primary producers, pathogens, arthropods and other biotic communities. The abiotic environmental compartments include animal housing and associated areas and materials, farm systems, machinery and light equipment, feed and water, climate variables and farm waste. Various interactions connect those components. Health management acts upon ecosystem components and their interactions and is a principal means through which humans deliberately modify the farm ecosystem; it is based on human decisions, which influence multiple components of the ecosystem and direct the interactions within the system. Health management can modify interactions throughout the farm ecosystem, with effects that may extend beyond their immediately intended targets. The consideration of these wider relationships can support better-informed and integrated health management of sheep and goat farms and their animal populations.

## Simple Summary

Sheep and goat farms include much more than their livestock. They also include people, other domestic animals, wildlife, vegetation, microorganisms, and the physical farm environment, all of which can interact with one another. This paper proposes considering small ruminant farms as managed ecosystems, in which these living and non-living components are connected through interactions and flows. The importance of individual components and their relationships can vary according to the type of farm and its management. Humans have a particularly important role because their decisions can modify the components of the farm ecosystem and the interactions between them. Health-management practices, including vaccination, parasite control, biosecurity, nutrition and milking management, can therefore have effects extending beyond their intended targets to other parts of the farm ecosystem. Considering these wider relationships can contribute to more integrated health management and a better understanding of the consequences of management decisions on sheep and goat farms.

## 1. Introduction

### 1.1. Background

Ecosystems refer to biological communities of interacting organisms and their physical environment, which creates and involves a complex network or interconnected system. They are formed by organisms in interaction with their environment [1] and consist of all the living organisms therein and the abiotic pools (or ‘physical environment’) with which these organisms interact [2,3,4]. Ecosystems are characterized by four elements: (i) a biotic component, (ii) an abiotic complex, (iii) the links and interactions within and between these, and (iv) the physical space that they occupy.

Agricultural ecosystems have been defined as ‘the ecosystems in which humans have exerted a deliberate selectivity on the composition of the biota, i.e., the crops and the livestock maintained by the farmer, replacing to a greater or lesser degree the natural flora and fauna of the site’ [5]. Significant characteristics of agricultural ecosystems are their management by humans and their use for provision of various goods and services to the human population. These, primarily, are related to and intended to cover food requirements. Their structure and function are determined by biological and environmental processes and also by the relevant human interventions and decisions.

Ecosystems are dynamic entities, as, periodically, they can be subjected to disturbances and changes; thus, they are consistently in the process of adaptation and recovery from these, in order to restore the equilibrium therein. Agricultural ecosystems are manipulated and modified with greater intensity and more frequently than natural ecosystems, which results in reduced biological diversity within the systems [6]. These modifications arise from the various management practices applied in agriculture [7].

Animal farms are a distinctive type of agricultural ecosystem, in which livestock interact continuously with other living organisms (e.g., people, other animals, pathogens) and with the physical environment of the farm under the influence of human management. Importantly, the management decisions applied on the farms can affect the interactions between the components of a farm ecosystem.

### 1.2. Overview of Production Types and Management Systems in Small Ruminant Farms

Small ruminant farms vary substantially in their production types and management systems, which can influence the components, interactions, and flows occurring within their ecosystems.

The main production types of small ruminant farms refer to meat production, dairy production, wool or fiber production, pelt production, and production of reproductive material (animals, semen, or embryos). It is noteworthy that on many occasions, although there is a leading production type on a farm, production also involves additional types as well. For example, there is some wool production in all types of farms, as, in most cases, sheep may require shearing at annual intervals; further, there is meat production in dairy farms, as a lactation period follows the birth of lambs or/and kids, which would grow and need to be sold.

The main management systems have been eloquently described in a paper by the European Food Safety Authority [8], in which six distinct management systems have been described, specifically: shepherding, intensive system, semi-intensive system, semi-extensive system, extensive system, very extensive system (Table S1 [8]). There are also farms following mixed management systems, which depend on the annual cycle applied on the farms, for example, a seasonal mix of semi-intensive management (during the winter) and semi-extensive (during the summer) [8]. There are also subdivisions of these management systems; for example, the shepherding management type includes three sub-classes: nomadic, transhumant, and agropastoral systems [8,9].

### 1.3. Scope of the Review

The objectives of this paper are the proposal of a narrative conceptual framework in which small ruminant farms are considered as managed ecosystems and the description of a basis for developing health management of small ruminant farms within this framework. The paper presents the identification, definition, and characterization of the principal biotic components and abiotic compartments, the examination of interactions occurring within and between these components, and the description of the role of health management in modifying interactions within these systems. In this model, small ruminant farms are viewed as dynamically managed ecosystems composed of interconnected biotic components and abiotic compartments, rather than simply as populations of animals maintained within a defined area. Accordingly, farms can function as networks of ecosystem interactions, wherein various, often complex, interactions take place.

## 2. Methodological Approach

### 2.1. International Ecosystem-Related Literature on Small Ruminants

Initially, in order to assess the extent and the development of ecosystem-related concepts and terminology in the international literature on small ruminants, a topic search of the Web of Science database was performed using the terms [sheep OR ovine OR *Ovis aries* OR goat* OR caprine OR *Capra hircus*] AND [ecosystem*] (the latter search term encompassing published papers, in which ecosystem-related concepts and terminology were used in various contexts, not necessarily indicating consideration of small ruminant farms as ecosystems) identified 2650 articles and reviews published up to the end of 2025.

**Figure 1:**
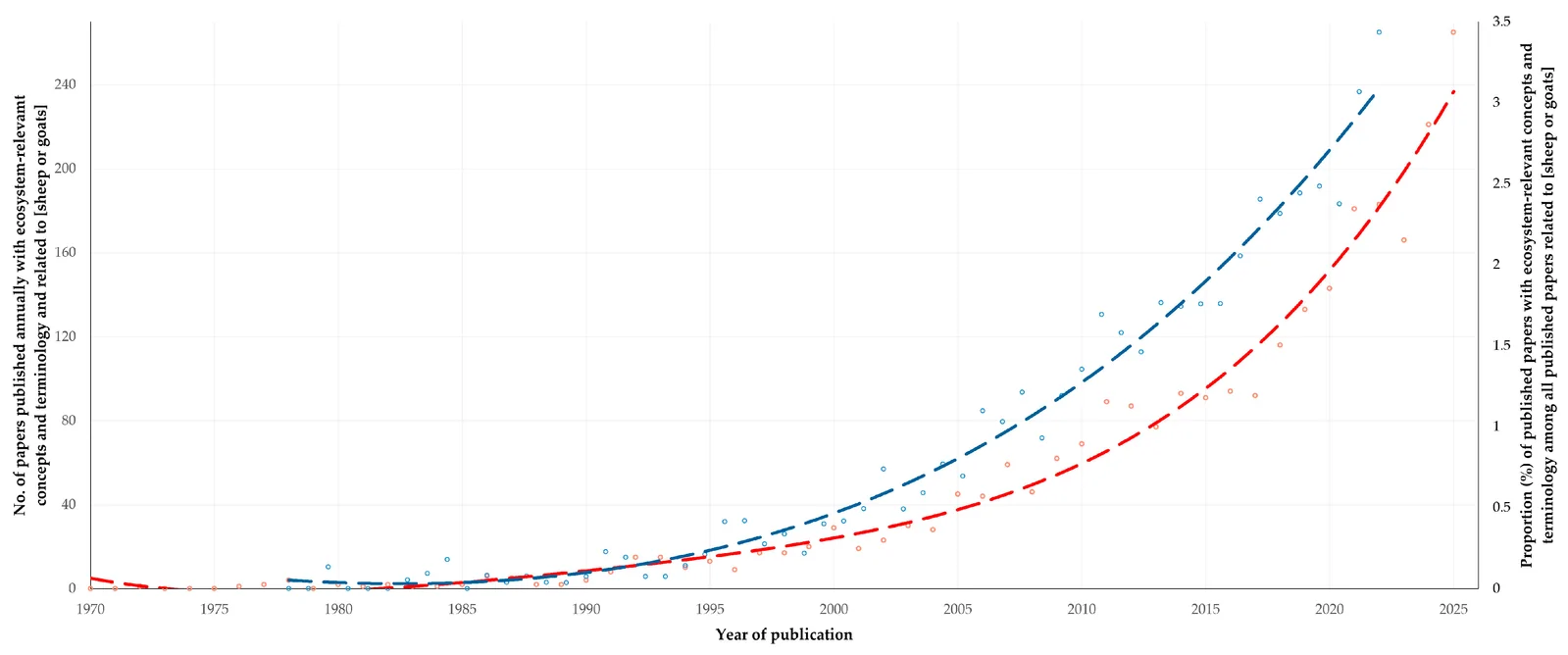
Scatter plot of the number of papers published annually related to sheep or goats with ecosystem-relevant concepts and terminology (red dots) and the proportion (%) of such papers published annually among all papers published related to sheep or goats (blue dots), from 1970 to 2025 (dashed lines are trendlines).

Overall, the use of ecosystem-relevant concepts and terminology in papers related to small ruminants has progressively increased, particularly during the last decade. Of the above published papers, 60.3% were published during the period 2016–2025. Moreover, the proportion of papers with ecosystem-relevant concepts and terminology among all publications concerning sheep or goats [10] increased from 0.7% during the period 2001–2005 to 1.6% during 2011–2015 and 2.8% during 2021–2025 (Figure 1). These findings indicate the increasing use of ecosystem-relevant concepts and terminology in small ruminant research.

The records retrieved through this search also provided a principal source for identifying ecosystem-related publications considered in the development of the conceptual framework and the writing of this review.

### 2.2. Development of the Conceptual Framework

The records retrieved through this search also provided a principal source for identifying ecosystem-related publications considered in the development of the conceptual framework and cited in the review.

The framework was developed as a conceptual synthesis based on the authors’ clinical and research experience and long-standing work in the field of small ruminant health management, together with established principles of ecosystem theory. Accordingly, the proposed framework is qualitative. This paper is intended to characterize the principal components and compartments, interactions and flows, and their relationships, rather than to quantify the magnitude of individual flows.

The proposed framework is intended to complement existing One Health, agroecosystem, socio-ecological, and herd-health perspectives by considering the individual small ruminant farm as its principal unit of analysis. The specific focus refers to the systematic organization of biotic components and abiotic environmental compartments of the small ruminant farm ecosystem, in accordance with their interactions and flows, directionality, functional boundaries, and human management and feedback.

### 2.3. Literature Search and Selection

Literature supporting the conceptual framework and the examples presented in the review was identified through complementary approaches. Ecosystem-related publications relevant to small ruminant research were identified principally from the Web of Science search described above. The Web of Science database was also used to identify published papers relevant to conceptual domains considered, outside the remit of small ruminant work, by using search terms selected according to the specific conceptual domains and topics addressed in the respective sections of the paper, as outlined above, within a search period from 25 March to 27 September 2026. Moreover, targeted searches were performed in order to identify authoritative sources required to support specific aspects of the framework, including documents and information published by international and European organizations, regulatory authorities, and other official bodies. In addition, foundational and seminal publications relevant to ecosystem theory and other concepts considered in the review were also included, irrespective of their retrieval through the bibliographic searches. Finally, additional relevant publications already known to the authors through their previous clinical and research work in small ruminant health and production were considered, where directly relevant to the concepts or examples discussed.

Records identified through these approaches were assessed for relevance to the conceptual domains considered in the review. Publications were retained when they provided information concerning the identification or characterization of biotic components or abiotic environmental compartments of small ruminant farms, of the interactions or flows among these, of farm boundaries and external drivers, or of ecosystem-level consequences of health-management interventions. As the objective of the paper was the conceptual synthesis rather than the estimation of pooled effects, study quality was not quantitatively scored, and no meta-analysis was performed.

Accordingly, the literature informed and supported the development of the framework, but the framework, as a product of the authors’ clinical and scientific experience and conceptual analysis, was not derived through a formal systematic evidence-synthesis procedure.

## 3. Small Ruminant Farms as Ecosystems

### 3.1. Conceptual Framework and Elements of the Ecosystems of Small Ruminant Farms

Within the proposed approach, the ecosystem of small ruminant farms can be considered to be characterized by five principal elements relevant to the concept. These are as follows:

- Components and compartments.
- Interactions and flows.
- Directionality of the interactions and flows.
- Inputs and outputs crossing the physical or functional boundaries of the system.
- Human management and feedback.

#### 3.1.1. Biotic Components and Abiotic Environmental Compartments

This approach includes the biotic components of the ecosystem (Table 1), which participate functionally, to a greater or smaller degree, in the interactions that occur within the farm ecosystem. Moreover, it includes the abiotic environmental compartments (Table 2), which comprise the non-living physical components, resources, and conditions of the farm environment that interact with the biotic components and participate in interactions within the farm [11,12,13].

#### 3.1.2. Interactions/Flows and Directionality

Within this framework, there may be various interactions and flows. Interactions represent functional relationships between components or/and compartments of the ecosystem, and derive from animal–human or animal–animal interactions or competition; examples among these include grazing, predation, infections, and utilization of environmental resources. The nature and intensity of these interactions can vary according to the production type and management system applied, and, ultimately, all these can influence the health of small ruminants [14,15]. Further, flows represent movements or transfers of entities or materials into, within, or out of the ecosystem; such examples refer to animals, people, pathogens, feeds, pharmaceutical products, or farm waste.

The directionality of interactions and flows within the farm ecosystem varies according to the components involved and the nature of their interaction [16]. Some flows are predominantly unidirectional, like the transfer of nutrients from feed to livestock, the export of animal products from the farm, or the livestock predation by wildlife; for example, feed administered to livestock provides nutrients for animal products (e.g., milk) and also contributes to the production of excreta, forming farm waste and manure, which then can be applied to the farm grazing paddocks. Other interactions can be bidirectional, for example, the transmission of pathogens between livestock and wildlife sharing grazing areas or between livestock and humans (i.e., dissemination of zoonotic pathogens) or interactions between livestock and vegetation, which provides nutrients to livestock through grazing, whilst livestock can modify the composition and availability of vegetation through selective grazing and trampling. Additionally, some flows can form cycles within the ecosystem [17]; for example, nutrients consumed by livestock through grazing are partly returned to the soil through excreta and may subsequently be taken up by vegetation and thus become available again to grazing animals.

In this respect, it is noted that health management can modify the direction, the magnitude or the continuity of these interactions and flows. There are several means by which this can be achieved, for example, control of animal movements, grazing management, biosecurity measures or management of farm waste.

#### 3.1.3. Inputs/Outputs

Inputs into the system would include (among others) purchased replacement animals, feed, water, pathogens and veterinary products, as well as people visiting the farm. Outputs from the system would include (among others) animal products, waste, pathogens, as well as people from the farm visiting other farms. Some of these can be directed by the various management decisions (e.g., the purchase of feed or replacement animals); others, however, occur independently of such decisions, although in many cases they can be controlled by relevant decisions (e.g., incursion of predator animals through setting up fences in the physical borders of the farm or the introduction of pathogens through maintaining a quarantine process in purchased animals).

#### 3.1.4. Boundaries

The physical boundaries of the farm can be considered to coincide with the farm premises. Nevertheless, its functional boundaries may extend beyond the premises; as the biotic components move, they can establish connections and interactions with the physical environment, as well as with biotic components of other farms [18]. Relevant examples include livestock grazing outside the farm on grazing lands shared with livestock from other farms and with wildlife, arthropod vectors moving in and out of the farm, and humans moving between farms. This supports the idea that the functional boundaries are determined spatially, and also by interactions of the components connecting the farm ecosystem to the surrounding environment.

The distinction between physical and functional boundaries [19] becomes particularly evident in farms managed under the semi-extensive or extensive management system, as well as in transhumant systems. In these circumstances, livestock would leave the physical premises of the farm and use grazing areas and water sources shared with livestock from other farms and with wildlife. Thus, although the livestock remain part of the population of a particular farm, they may temporarily participate in interactions occurring within environmental compartments located outside the physical boundaries of that farm. The relationship between physical and functional boundaries would vary in accordance with the typology of the farm. For example, in an intensively managed farm, livestock would not normally graze, thus physical and functional boundaries would, to a large degree, coincide; in a non-transhumant extensively managed farm, the functional boundaries would differ on a daily or weekly basis, in accordance with the locations selected for grazing by the farmer on particular days or weeks, whilst remaining centered at the farm premises.

In contrast, in a transhumant system, the farm may be seasonally established at different locations [20,21], with corresponding shifts in its physical (and consequently functional) boundaries, according to the seasonal locations of the flock or herd and its associated camp. In transhumant systems, the seasonal livestock movements can connect distinct environmental settings and abiotic environmental compartments, with the livestock population providing continuity between them.

Moreover, where communal grazing areas are used by several flocks or herds, interactions involving grazing resources and pathogens cannot be attributed exclusively to a single farm ecosystem, and relevant management decisions made by one farmer may affect animals of other farms. Transhumance, in particular, can ‘connect’ otherwise spatially separated farm ecosystems through movements of livestock and interactions associated with these. Hence, one can consider that the functional boundaries of a small ruminant farm ecosystem are dynamic, permeable and seasonally variable, and may possibly overlap with those of other farm ecosystems.

It is notable that, under such circumstances, health-management practices applied in flocks or/and herds, for example, biosecurity measures or endoparasite control, may require some coordination between farmers sharing the same grazing resources. Lack of such coordination may lead to a ‘dilution’ of the potential beneficial effects of the health-management interventions applied to flocks or/and herds involved.

#### 3.1.5. Human Management and Feedback

**Figure 2:**
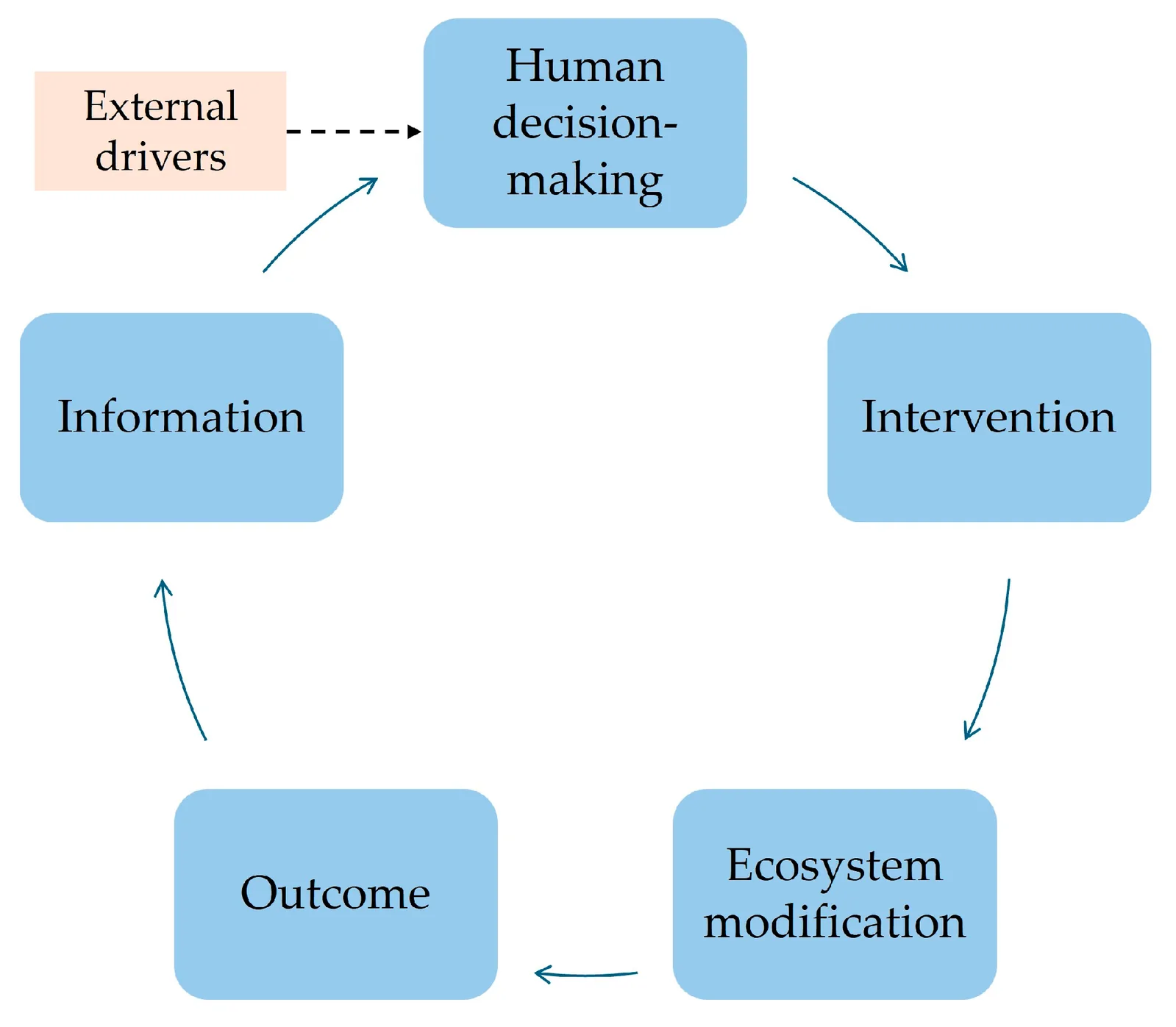
Schematic general description of the feedback loop created through the implementation of decisions related to health management applied on farms and the monitoring of their outcomes. Human decision-making is influenced by external drivers (e.g., economic, regulatory, societal, labor-related), as well as by information derived from ecosystem outcomes, which also provides feedback for further management decisions (full arrows indicate interactions occurring within the ecosystem, dashed arrow indicates external influences).

Accordingly, any small ruminant farm can be characterized within this framework by identifying its biotic components and abiotic compartments, the interactions and flows connecting them and their directionality, the inputs and outputs crossing its functional boundaries, and, finally, the human decisions and feedback processes that modify the system. A schematic description of the feedback loop created through the implementation of decisions related to health management applied on farms is in Figure 2.

The farms are also characterized by the health management applied therein. Farm management is the broad human-directed process taking place on farms; health management is an important subsystem of the overall farm management. This refers to a variety of interventions and tools, for example, animal replacement policy, biosecurity measures, formulation of rations, milking routine, anthelmintic treatments, vaccinations, all of which can contribute to modifying the characteristics of the components and the interactions between them. Outcomes (related to animal health and welfare and animal production) resulting from the application of the various interventions can influence subsequent decisions on the health management of farms.

A relevant specific example of the above refers to the administration of the anthelmintic treatment considered to be appropriate in a case of parasitic infection; this would potentially lead to control of the infection and, consequently, to an increase in animal production. Based on observations regarding animal production outputs (e.g., milk yields or growth of young animals), as well as on the information obtained from specific laboratory tests (e.g., the parasitological examination of fecal samples), a new decision can be taken subsequently as to whether further treatment might be required.

#### 3.1.6. Integrated Application of the Framework

Overall, the elements described above can be applied systematically to the principal interactions and flows occurring within small ruminant farm ecosystems. Table 3 presents representative examples and also indicates how their characteristics may vary according to production type, management system, and geographical setting of small ruminant farms.

As an illustrative example of the framework at farm-ecosystem level, a transhumant sheep flock and an intensively managed dairy goat herd can be contrasted. In the former, livestock would interact seasonally with changing vegetation, soil, water resources, wildlife and livestock from other farms, with functional boundaries shifting geographically and overlapping with those of other farm ecosystems through communal grazing. In the latter, livestock, humans, pathogens and microbiota represent major biotic components interacting with housing, milking parlor, concentrated feeds and sources for animal watering as important abiotic environmental compartments; replacement livestock and feed would enter into the system across clearly defined physical boundaries, whilst milk, kids and waste would constitute major outputs from the system. Moreover, in the former, interactions and health-management measures would depend to some extent on decisions involving multiple farmers and shared environmental compartments; in contrast, in the latter, management would operate principally within stable and defined physical boundaries. In this instance, the application of the same framework reveals differences in ecosystem structure, interactions, boundaries and management constraints, which might not become readily apparent from consideration of the livestock component on its own.

### 3.2. External Drivers

Factors outside the farm ecosystem (‘external drivers’) may influence decisions upon which health management in small ruminant farms is based. They include financial considerations (e.g., market prices and cash flow), legal and regulatory requirements and policies, societal preferences and pressures, availability of human resources, and climate change. Although these factors are not components or compartments of the farm ecosystem, they can modify its structure and function indirectly through their effects on management decisions.

Within this context, economic, policy, regulatory, and societal conditions can influence stocking rates on farms, feeding and grazing strategies, animal replacement approaches, investments in infrastructure, and implementation of health-management measures. For example, within the European Union, interventions implemented through the Common Agricultural Policy (CAP) under Regulation (EU) 2021/2115, which establishes the framework for the CAP Strategic Plans of Member States, can influence the economic viability and management of small ruminant farms and production systems; additionally, regulatory requirements can directly influence management options, through provisions concerning animal identification, movements and transportation, and disease surveillance and control (Regulation (EU) 2016/429), the use of veterinary medicinal products (Regulation (EU) 2019/6) and the manufacture and use of medicated feed (Regulation (EU) 2019/4).

Additionally, specifications associated with protected-designation products may impose additional constraints on farm management. This is particularly relevant in small ruminant dairy production, as several major European cheeses, for example, ‘roquefort’ in France, ‘feta’ and ‘graviera Kritis’ in Greece, ‘pecorino romano’ in Italy and ‘queso manchego’ in Spain, are registered as Protected Designations of Origin (PDO). Moreover, in the United Kingdom, various meat products, for example, the ‘Orkney lamb’, ‘Shetland lamb’ or ‘Welsh lamb’, have also received protected status, making such specifications relevant to sheep-meat production as well. The respective product specifications include requirements concerning the geographical origin of production, the origin or breed of the animals, the type of milk used or other aspects of production. Such requirements can influence decisions concerning animal breeds, feeding and production practices and can thus act as external drivers of management decisions at farm level.

The economic situation, which includes and refers to prices of feed and other inputs into the farm, prices of animal products, availability of capital and labor costs, can also influence decisions regarding feeding, replacement of animals, stocking rates, investments in housing and equipment and the implementation of preventive health measures. A specific example refers to reduced cash flow occurring during the second half of gestation (i.e., when no offspring are available for sale and females are not being milked), which may contribute to the purchase of limited amounts of concentrate feeds; this, coupled with harsh weather conditions during the winter, limiting grazing by pregnant females, can increase the risk of development of pregnancy toxemia in sheep flocks; severe cases may result in the death of affected ewes, thereby modifying the livestock component of the ecosystem.

Economic considerations can also influence the structure of the livestock component and the flows of animals and products from the farm ecosystem. For example, in Mediterranean dairy sheep systems, including those in Greece, lambs are commonly slaughtered young, producing light carcasses, whilst milk constitutes the principal product of the production system [22]. Thus, light lamb carcasses (approximately 9 to 12 kg) are produced after a suckling period of up to two months [23,24]. This production pattern reflects the economic importance of milk production together with a market demand for light lambs and results in the early removal of young animals from the farm ecosystem. In contrast, in production systems oriented towards heavier lamb carcasses, offspring remain within the farm ecosystem for a longer period, thereby influencing the age structure of the livestock component and the duration of their interactions with other ecosystem components.

More generally, economic considerations by farmers can influence health-management decisions. Farmers weigh the expected costs of preventive or therapeutic interventions against the anticipated benefits of such interventions, in terms of reduced disease losses and improved animal production, thereby affecting the interventions applied within the farm ecosystem.

Availability and cost of labor represent further external constraints, particularly in management systems requiring substantial animal supervision or handling (e.g., milking). The trend for an increasing age of farmers can become a potential limiting factor for the availability of agricultural workforce in the future; for example, in Europe, farmers younger than 40 years manage only 12% of holdings [25]. Currently, the employment of foreign workers contributes to addressing labor shortages and sustaining farming activities in various countries [26,27,28].

In this way, external drivers may affect inputs into and outputs from the farm, the number and composition of livestock, contacts between biotic components and the use of abiotic environmental compartments. Their effects can consequently propagate through several components and interactions of the farm ecosystem, although the external drivers themselves do not constitute components or compartments of that ecosystem.

### 3.3. Abrupt Ecosystem-Scale Perturbations

Small ruminant farm ecosystems may occasionally be subjected to abrupt perturbations, which can substantially modify their components, interactions, and flows. Occasionally, these may potentially result in partial or complete disruption of the ecosystem. Such perturbations can have different origins.

Major origins of perturbations include the incursion of an epizootic pathogen and environmental events, for example, floods, wildfires, or earthquakes. In the former, the initial perturbation originates from the introduction of a pathogen, which principally affects the core component of the ecosystem, the livestock, and its interactions, whence its direct effects and the subsequent human responses may propagate throughout the ecosystem. In contrast, environmental-origin perturbations can act simultaneously on multiple biotic components and abiotic compartments of the ecosystem, potentially producing immediate system-level disruption and, in extreme cases, extensive destruction.

#### 3.3.1. Incursions and Control of Epizootic Infections

The introduction of pathogens, which may take place in a variety of ways, for example after predation attacks (e.g., rabies), after purchase of subclinically infected livestock (e.g., peste des petits ruminants), after the visit of a vehicle that had previously been on farms with infected animals (e.g., sheep pox) or by airborne transmission (e.g., foot-and-mouth disease), represents an input crossing the boundaries of the ecosystem and may result in infection of the livestock population, with subsequent effects on animal health. Depending on the characteristics of the disease, pathogen dissemination may also involve other biotic components of the ecosystem, including humans, other domestic animals on the farm, or wildlife. Thus, an epizootic infection would possibly modify several interactions and flows within the farm ecosystem concurrently.

The occurrence of such an incursion can also trigger substantial changes in ecosystem management. Depending on the infection and the regulatory framework, control measures would include restrictions on animal movements, isolation of infected or exposed animals, or compulsory vaccination. For particular infections, the regulatory framework may provide for compulsory culling or depopulation of affected farms [29].

Overall, these interventions modify the number and the composition of the livestock component and also interrupt or redirect movements of animals, people and animal products across the functional boundaries of the farm. Animal culling and depopulation of farms generate substantial flows of carcasses from the culled animals, which necessitate safe disposal. Where complete depopulation has been implemented, removal of the livestock population can be considered a partial dismantling of the affected farm ecosystem through the removal of its core biotic component.

The recent successive occurrence of three major epizootic infections in Greece provides a characteristic example. Initially, incursion by *Peste-des-petits-ruminants virus* (July 2024) was confirmed in the central part of the country and subsequently spread to several farms in other regions. This was followed by a still ongoing epidemic of *Sheep pox virus* infection that disseminated throughout the continental part of the country and some islands as well. Finally, an outbreak of *Foot-and-mouth disease virus* infection was confirmed on the island of Lesvos (March 2026) [30]. These successive incursions resulted in disease-control interventions affecting livestock populations, animal movements and farm activities across substantial parts of the small ruminant sector in the country.

Following successful control or eradication of an incursion, the ecosystem would undergo a period of recovery and reorganization. Animal movements, production activities and interactions with environmental compartments are progressively re-established. Where complete depopulation has taken place, subsequent restocking introduces a new livestock population and can therefore be viewed as reconstruction of the farm ecosystem.

In cases of epizootic infections concurrently affecting large numbers of farm ecosystems, their consequences may also propagate beyond individual farms and persist over time. A recent example is provided by the prolonged epizootic situation in Greece: reductions in the small ruminant population as a consequence of depopulation have contributed to reduced availability of sheep milk and consequent pressure on feta cheese production. In September 2026, a representative of the Greek dairy sector quoted in a press report indicated that the national feta stocks might not suffice until the end of the year and estimated that retail prices of the product could increase by 15% to 25% [31]. Thus, perturbations originating within multiple farm ecosystems can propagate through downstream production and market systems, generating consequences beyond the farms in which the initial perturbations occurred.

This example illustrates the dynamic nature of the proposed framework: an external biological input (epizootic pathogen) leads to a progressively propagating, ecosystem-wide perturbation. The case involves the modification of multiple components, interactions, flows, and boundaries before potential reorganization.

#### 3.3.2. Environmental Perturbations

Abrupt perturbations of the farm ecosystem may also result from environmental events [32]. Floods, wildfires, earthquakes, severe storms and other extreme events can simultaneously affect several biotic components and abiotic environmental compartments. Their immediate effects may include mortality or displacement of livestock and other animals (e.g., wildlife moving out of their natural habitats and re-establishing nearer a farm), destruction of vegetation, contamination or loss of feed and water resources and damage to farm infrastructure, systems and equipment. Such events also interrupt flows of animals, people, feed and animal products into and out of a farm and may temporarily or permanently modify its physical boundaries.

The magnitude of these effects can range from temporary disturbance of particular components or interactions to extensive disruption of the farm ecosystem, potentially extending to complete dismantling and destruction. In such cases, recovery requires restoration of infrastructure, replacement of equipment and feed resources and, if substantial livestock losses have taken place, introduction of replacement animals. In extreme circumstances, where the livestock population and substantial parts of the physical farm environment have been lost or destroyed, subsequent recovery can be viewed as a reconstruction of the managed farm ecosystem.

## 4. Biotic Components of the Ecosystem of Small Ruminant Farms

### 4.1. Livestock: Sheep or/and Goats

In an ecosystem approach to small ruminant farms, sheep or/and goats constitute the core biological component, around which the farm ecosystem is organized. The livestock population on a farm is a dynamic component of the farm ecosystem. Its composition can vary over time, whilst the animals consume resources and generate products and waste. Livestock influence and interact with the other components of the farm ecosystem.

The specific characteristics of these animals should be considered, as several aspects of health management depend on their particularities. For example, differences among breeds of sheep or/and goats in resistance to diseases have been documented [33]; further, animals of different ages have varying nutritional needs and requirements for space within farm buildings.

The production stage of the animals is important for their interaction with other components of the ecosystem and for the application of the appropriate health management. The needs and requirements of sheep or/and goats differ considerably among the mating season, the late pregnancy stage, the suckling phase, and the milking period [34,35]. During each of these stages, animals have varying nutritional requirements, contrasting physiological and immunological status and different needs regarding housing, feeding and veterinary interventions. Consequently, modifications in the annual production cycle on a farm can result in changes within the ecosystem and the pressure that livestock places on other components of the ecosystem [36,37].

Movements of sheep or/and goats constitute a significant part of interactions within the farm ecosystem and can alter the equilibrium therein. Animal inputs refer mainly to animal purchases for the needs of the farm (e.g., replacement animals); occasional transfer of animals from another farm may also occur, for example, the transfer of rams or/and bucks during the mating season, although such movements pose significant threats for pathogen dissemination into the ecosystem and must be discouraged. Animal outputs include animals sent to abattoirs, animal sales to other farms, and animal deaths. In all cases, these influence the farm ecosystem, because they modify livestock numbers and concentrations, as well as the requirements for resources for the livestock. These changes also potentially necessitate changes in the health-management programs applied on farms, for example, the implementation of biosecurity measures or the modification of nutritional management to cover the new entrants into the farm [38,39].

### 4.2. Other Domestic or Synanthropic Animals on Farms

Beyond sheep and goats, various animal species (domestic or synanthropic) can be present within the ecosystem of small ruminant farms. For some, their presence is intentional, as they are used to perform specific functions within the farm or are maintained for production purposes; for others, their presence is incidental (synanthropic species). These animals can interact to varying degrees with livestock, people, and the farm environment and constitute additional biotic components of the ecosystem. These animals hold diverse functional roles within small ruminant farms. Most of them are included in the ecosystem to provide services or additional agricultural products, whereas others among these animals may intrude and compete for resources or can cause damage. All these animals establish additional links among the components and compartments of the ecosystem and contribute to its complexity and connectivity.

Since antiquity, dogs have served as guardians and herders in livestock farms [40]. Farmers maintain dogs for their contribution to protection against wildlife predators that may attack sheep and goats (‘guardian dogs’); dogs can also assist in the management and movement of livestock (‘herding dogs’) [41,42,43]. Guardian dogs interact with animals or people entering the farm ecosystem, particularly with those perceived as potential threats, whilst herding dogs interact strongly with livestock within the ecosystem. Dogs also interact closely with people on the farm, and a complex ethno-ethological relationship between the farmers and the dogs has also been described [44]. These animals occupy a particular position within the farm ecosystem, forming links between livestock, people and the surrounding environment.

A variety of other farm animals (cattle, pigs, rabbits, poultry) can be present on small ruminant farms [45,46]. These may include animals present for commercial farming reasons, as is the case in large commercial multi-species operations, or animals available to provide food for the farmer and the family (e.g., fattening calves or pigs). Their presence increases biological diversity on farms and generates additional requirements for feed, water, housing, and labor; also, it increases the potential for inter-species transfer and dissemination of pathogens. All these contribute to expanding the diversity of animal interactions within the farm ecosystem.

Horses and other equids may also be present on small ruminant farms. These can be used in free-riding activities, as well as for work purposes (e.g., transportation or other activities associated with management of farms). Their role within the farm ecosystem therefore would differ, in accordance with the purpose of their maintenance and the degree of interaction with livestock and people.

Cats are also frequently present on small ruminant farms. Traditionally, an important role has been the control of rodent populations within and around the farm grounds, within farm buildings and feed-storage areas. Nevertheless, cats may also command considerably large areas outside the farm boundaries, establishing an interface between the farm and its surrounding area and environment [47]. Anecdotal reports have also indicated the possible development of social bonds between cats and sheep on the farm.

Cats and dogs contribute to the dissemination of the abortifacient protozoa *Toxoplasma gondii* and *Neospora caninum*, respectively, to sheep or/and goats [48,49,50,51]. This pattern of dissemination is stronger in intensively or semi-intensively managed farms, wherein provision of concentrated feed or hay (potentially contaminated with the above pathogens through the feces of the carnivores) is an integral component of the management system. The pathogenetic effect of these two protozoa varies according to the stage of pregnancy at which infections have occurred [48,49,50,51]. This suggests that interactions between biotic components of the farm ecosystem are affected simultaneously by the management system and the production stage of livestock.

Synanthropic rodent species constitute another biotic component of farms, although they are unwanted on farms. Principal species include brown rats (*Rattus norvegicus*), field mice (*Apodemus sylvaticus*), house mice (*Mus musculus domesticus*) and roof rats (*Rattus rattus*) [52]. Rodents consume feed available for livestock and can also contaminate large quantities of feed with urine and fecal excreta; they also cause damage to components of farm infrastructure and equipment (e.g., wooden structure, plastic insulation material, electrical wiring) [52]. Finally, rodents can also be responsible for the introduction and dissemination of pathogenic microorganisms (e.g., *Leptospira* spp.) into the farm ecosystem; several of these pathogens are of zoonotic importance, which indicates an interaction of a synanthropic species with humans in the ecosystem [53].

### 4.3. Wildlife

Wildlife animals are not directly managed components of small ruminant farms; they live and originate from outside the premises of farms. They are nevertheless connected with farms and contribute to the wider farm ecosystem. Their connections arise mainly, because sheep and goats often share grazing areas and other natural resources with wildlife, and also because predatory wildlife can make incursions into farms. Wildlife populations are highly mobile and move independently of regional or national boundaries and can provide a dynamic and perennial interface between small ruminant farms and the surrounding natural environment. Interactions between livestock and wildlife occur mostly after the incursion of the latter species into the farm. However, interactions can also occur with no direct contact, through shared use of the same grazing grounds, as well as by animals living on the farm preying outside the physical premises of the farm.

Shared use of grazing areas by sheep or/and goats and wildlife ruminants is a common example of this interface. Across different geographical regions, relevant wildlife species include Asian ibex (*Capra sibirica*) in Asia, bighorn sheep (*Ovis canadensis*) and elk (*Cervus canadensis*) in North America, chamois (*Rupicapra rupicapra* and *R. pyrenaica*), European mouflon (*Ovis aries musimon*), red deer (*Cervus elaphus*), European and Siberian roe deer (*Capreolus capreolus* and *C. pygargus*) in Europe or/and Asia) and white-tailed deer (*Odocoileus virginianus*) in the Americas. This shared grazing can result in competition for common resources, particularly vegetation on grazing land and water. The shared use of these areas provides opportunities for pathogen exchange between livestock and wildlife animals, for example, gastrointestinal trichostrongylids [54,55,56,57] or abortifacient bacteria [58,59,60]. The shared grazing areas represent an important spatial interface linking the farm ecosystem with wildlife populations and the wider natural ecosystem around the farms.

Another important interaction between wildlife and small ruminant farms refers to predation. The principal species of wildlife predators for sheep or/and goats vary between geographical regions (Table S2 [61,62,63,64,65,66,67,68,69]). The predation of livestock has generated significant human–wildlife conflicts. Livestock losses inflicted on farms have led farmers to hunt wildlife populations in their attempt to minimize predation risk [61,66,70,71]. This highlights that potential interactions between livestock and wildlife can extend to interactions between wildlife and people (i.e., two non-core components of the farm ecosystem), with potential consequences for wildlife populations and conservation. Further, it indicates that interactions between ecosystem components may extend beyond the boundaries of the farm.

The effects of predators on livestock are not limited to direct attacks. The conceptual framework of ‘landscapes of fear’ defines potential predation risk as perceived by prey. This is a behavioral characteristic and may be defined at individual animal or animal population-level; it depends upon the sensory modalities of the prey [72]. The principal factor influencing animals in the development of ‘landscapes of fear’ is the predation risk [73,74].

Predation can be controlled or prevented through the use of livestock guardian dogs [75,76,77]. Their presence creates an additional interaction between wildlife, livestock, dogs and farmers within the farm ecosystem. Interactions between guardian dogs and wild carnivores may have consequences beyond predator deterrence. An association was reported between the presence of wild canid predators near sheep farms and the detection of *Uncinaria/Ancylostoma* spp. and *Toxocara canis* in fecal samples from farm dogs [43]. These observations indicate further interactions between two non-core biotic components of the small ruminant farm ecosystem, i.e., farm dogs and wildlife mammals.

An important source of infection of farm cats with *T. gondii* is considered to be wild rodents infected with the organism, as studies showed that, in mice, *T. gondii* infection can be passed vertically from generation to generation [78]. Infected cats would then shed oocysts and contaminate the farm environment, leading to subsequent infection of livestock. This indicates an interaction between two non-core components of the farm ecosystem, which subsequently establishes an indirect link between wildlife outside the farm and farm livestock.

Reptiles include further wildlife animals interacting in small ruminant farms. Some species can act as predators for lambs or/and kids, whilst others can also contribute to the dissemination of pathogens to livestock [79,80]. Nevertheless, reptiles need not necessarily lead to adverse consequences for livestock farming. A recent study performed in Australia revealed a potentially beneficial ecosystem function related to heath goannas (*Varanus rosenbergi*): through scavenging of livestock carcasses, these lizards may contribute to carcass removal and potentially contribute to reducing populations of blowflies infesting sheep [81].

Wild birds can also use farm buildings, feed stores, troughs, manure, and pasture. In this context, wild birds cross the physical farm boundaries and interfere with its ecosystem.

It is noteworthy that the interactions of wildlife within the farm ecosystem depend on the management system applied to farms. Predation occurs more often in farms managed under the extensive or semi-extensive system, likely because livestock are less protected and consequently more exposed to interactions with wildlife. Farms managed under these two systems may include a higher number of dogs, in order to improve livestock protection, which again shows that farm management system plays a role in the interactions occurring within the farm ecosystem.

### 4.4. Humans

Humans differ from other biotic components of the farm ecosystem, because they have a dual position therein: first, they manage the farm, and they can modify the ecosystem to a smaller or greater degree; and, second, they constitute themselves a biotic component of the ecosystem, interacting with livestock, other animals, pathogens and the abiotic compartments.

There is a differentiation in the degree of interaction of humans with the ecosystem, which must be distinguished. First, farmers, family, and farm workers, who have continuous contact and interaction with the ecosystem; second, veterinarians, technical personnel, people in supporting roles (e.g., drivers of lorries that transport products from the farm) and other farmers, who have intermittent contact with the ecosystem, and also an intensive contact with multiple farms and animals; third, occasional visitors and hunters, who have a transient interaction with other farms and animals.

Certainly, the most significant manipulations in the ecosystem are performed by farmers. Farmers have the general responsibility for their farms and livestock and take decisions aiming to maximize production from animals on the farm, at the same time maintaining animal health and welfare [82].

Health management provides useful tools to farmers, in order to support decision-making. Further, farmers can involve other people in the decision-making process: they may consult veterinarians and other technical personnel, they can consider practices followed by other farmers in similar circumstances, and they can discuss their plans with members of their families. In this way, people other than farmers also take part in the decisions that lead to modification of the farm ecosystem.

Nevertheless, management decisions are not always correct and are influenced by individual characteristics of farmers. Corner-Thomas et al. [83] reported that, in New Zealand, farmers older than 60 years of age were using fewer health-management tools on their farms, including even anti-clostridial vaccination [83] (which is considered to be the core vaccination in small ruminant farms [84]) or ultrasonographic confirmation of pregnancy, which are tools considered to be essential in the health management of sheep or/and goat farms [35]. Such attitudes may have adverse consequences for the farm, particularly in the case of diseases and disorders requiring complex management for successful control and elimination, for example mastitis [85] or lameness [86]. The consequences of farmer-related characteristics would be subsequently mirrored in the productivity of farms and in product quality. In general, farm productivity has been found to decrease with farmers older than 45 years [87]. Farmer education can also play a role, as it guides relevant decisions and, consequently, farm productivity. In this respect, better education of farmers was reported to be associated with correct and prudent use and administration of antibiotics in sheep and goat farms [88], which in turn can contribute to successful treatment of infections and avoidance of development of antibiotic resistance.

People are also active components of the farm ecosystem. They can interact with animals and with the physical environment of the farm, and they are carriers of pathogens themselves; they can transfer pathogens into the ecosystem from outside sources or other similar ecosystems. Their involvement and association within the farm ecosystem, as described above, may affect pathogen introduction into the ecosystem. This involvement can also affect the exposure of people to the farm environment and consequently to potential zoonotic agents.

### 4.5. Vegetation

Vegetation on pastures grazed by livestock contributes substantially to the coverage of nutritional requirements of the animals. Nevertheless, sheep or/and goats may show preferences for particular plants or plant parts during grazing, which suggests that animals may actively select among available plant species and parts. This can be viewed as a livestock–vegetation interaction, which may influence animal nutrition [89,90,91]. Nevertheless, the importance of vegetation in small ruminant ecosystems is highly dependent on the management system applied on the farms; it is virtually non-existent or minimal in intensive or semi-intensive systems, progressively increasing in farms managed under the semi-extensive or extensive management systems.

Consumption of certain plants (e.g., *Veratrum californicum*) by pregnant ewes can result in embryotoxic effects, leading to congenital or developmental abnormalities in lambs [92]. Moreover, plants grazed by small ruminants may contain bioactive compounds with a potential direct effect on other components of the ecosystem; a notable example refers to the presence of tannins found in various shrubs (e.g., *Myrtus communis* (myrtle), *Quercus ilex* (holm oak), *Pistacia lentiscus* (lentisk)) or trees (e.g., *Schinopsis lorentzii* (red quebracho)), which have been found with anthelmintic effects against gastrointestinal trichostrongylids [93,94].

### 4.6. Pathogens and Arthropods

In small ruminant farm ecosystems, a variety of pathogens can affect livestock. These include prions, viruses, bacteria, fungi, protozoa, metazoan endoparasites and ectoparasites [95]. A non-exhaustive list of pathogens that affect sheep and goats is in Table S3. Infections or infestations of small ruminants by these pathogens can lead to various health problems and, consequently, to reduced production by affected animals. Pathogens constitute highly dynamic biotic components of small ruminant farm ecosystems. Their presence and abundance depend upon interactions with susceptible or carrier hosts, other animal species and environmental conditions, whilst these interactions are further influenced by the management practices applied on the farm. Consequently, pathogens and vectors can form links among multiple biotic components and abiotic compartments of the ecosystem, with potential consequences for animal health and farm productivity, as well as for public health.

Most of these pathogens have sheep or goats as principal hosts. Small ruminants can have active infections caused by these pathogens, or they may be carriers (e.g., *Staphylococcus epidermidis*, *Chlamydia abortus*). A general definition of an asymptomatic carrier refers to an infected animal that harbors an infectious agent without clinical signs of the associated infection and is capable of transmitting the microorganism to susceptible hosts [96]. Carrier animals can contribute to the transmission of pathogens during interactions with other components (e.g., susceptible animals) of the farm ecosystem. Of particular concern among pathogens are the zoonotic pathogens, which are defined as ‘pathogens that can be transferred from non-human animals to humans’ [97,98]. In contrast, a reservoir is a host population, or an epidemiologically connected set of host populations, in which an infectious agent can be maintained and from which it can be transmitted to a susceptible target population [99].

Some pathogens can be transferred to sheep or/and goats from other animal species that constitute their principal hosts. Examples of such organisms include *Brucella abortus* (from cattle) [100], *Erysipelothrix rhusiopathiae* (from pigs) [101], and *Streptococcus equi* subsp. *zooepidemicus* (from horses) [102]. These pathogen transfers highlight the importance of other animal species as interconnected components of the farm ecosystem.

Some arthropods that are pathogens of small ruminants themselves can also act as pathogen vectors. Vectors can be biological, which may carry pathogens that can multiply within their bodies and be delivered to new hosts (e.g., mosquitoes or ticks), or mechanical, which are organisms that play a purely mechanical role in transmission of pathogens (e.g., *Musca* flies transporting bacteria) [103,104]. These occupy two positions simultaneously within the ecosystem, i.e., pathogens and vectors. Notable arthropod vectors include ticks (vectors for, e.g., *Anaplasma phagocytophilum*, *Babesia ovis*, *Crimean-Congo hemorrhagic fever virus*) [105,106], biting midges of the genus *Culicoides* (vectors for, e.g., *Bluetongue virus*, *Orthobunyavirus schmallenbergense*) [107], mosquitoes (vectors for, e.g., *Rift Valley fever phlebovirus*) [108] and possibly ear mites (vectors for *Mycoplasma agalactiae*) [109]. Further, flies can act as mechanical vectors of various pathogens (vectors for, e.g., *Escherichia coli*, *Salmonella* spp.) [110].

Dung beetles (class Coleoptera, family Scarabaeidae) utilize livestock feces and contribute to dung decomposition and incorporation into the soil. Dung beetles can be seen as participating in nutrient cycling within grazing ecosystems [111]. That activity indicates their connection with soil, vegetation and farm waste. Dung beetle populations can be affected by residues of anthelmintic products administered to sheep or/and goats, which are excreted in the feces of treated livestock [112,113].

### 4.7. Other Biotic Communities

Microbial communities associated with livestock constitute an important component of the farm ecosystem. These communities occur at several anatomical sites of animals, for example, in the gastrointestinal tract [114,115], on the skin [116,117,118], as well as in the teat [119] and the mammary gland [120,121], and can influence nutrition, metabolism and health of colonized animals. The rumen microbiome is particularly important in ruminants, as its microorganisms participate in the degradation and fermentation of feeds, through which pathway dietary substrates are converted into compounds utilizable by the animal [122,123,124]. Methanogenic Archaea also contribute to flows of carbon from feeds through to production of methane [125]. Other microbial communities, for example those on the skin or in the mammary gland, interact with host tissues or with invading pathogens, and may be associated with maintenance of health or with changes occurring during disease. These communities therefore represent biotic components located within or directly associated with the livestock ecosystem.

Other microbial communities are associated with feed and stored feedstuffs and occupy a different functional position within the ecosystem. A characteristic example is the community of lactic acid bacteria involved in fermentation during silage production [126], through which microbial activity modifies the characteristics and preservation of feed to be subsequently consumed by livestock.

Distinct biotic communities also occur within environmental compartments of the farm. Soil contains a diversity of microorganisms, as well as various other organisms, for example mycorrhizal fungi, free-living nematodes and earthworms, which participate in decomposition, nutrient cycling and interactions with vegetation [127,128]. Their activity connects several parts of the farm ecosystem, particularly where nutrients contained in livestock excreta and manure are returned to soil and subsequently become available to vegetation. Consequently, these organisms constitute biotic communities with their own interactions and responses to environmental and management conditions.

All these communities, livestock-associated, soil-associated or feed-associated, can be modified, directly or indirectly, by human management. Some examples include the dietary changes altering rumen microbial communities and the potential development of relevant health problems (e.g., clostridial infections [129,130] or ruminal acidosis [131,132]) or the effects of antimicrobial or anthelmintic treatments on non-target organisms or communities associated with livestock [133,134]. The unintended effects of various management interventions on these communities are examples of propagation of actions towards one component or objective to non-target biotic components of the system and of the ensuing modifications in interactions and flows elsewhere therein.

## 5. Abiotic Environmental Compartments of the Ecosystem of Small Ruminant Farms

### 5.1. Overview

The abiotic environmental compartments of the farm ecosystem comprise the non-living resources, physical structures and environmental conditions that interact with and can influence the livestock and other biotic components of that ecosystem. Resources are distinguished as the various substances or objects in the environment, which are required by one organism and consumed or otherwise made unavailable for use by other organisms [1,135].

In small ruminant farms, the abiotic compartments of the farm ecosystem can differ greatly in accordance with the management system applied on the farms. For example, farms managed under intensive or semi-intensive systems are, on average, located at lower altitudes than farms managed under semi-extensive or extensive or very extensive systems [136]; there are differences in soil and vegetation, in accordance with the altitude of the location, which may be reflected in the area surrounding farm premises. Altitude differences were also found to be associated with varying climate variables [137,138], and this has also been reported with variables prevailing at the locations of farms [139]. Farms managed under the extensive or very extensive system can be low-input farms. Low-input farms intend to optimize the management and use of on-farm resources, at the same time reducing the use of production inputs from off-farm resources (e.g., fossil fuels, animal health products) [140,141]. Various aspects of the abiotic compartments of farm ecosystems vary across farms; for example, often there are no constructions (e.g., animal buildings) or systems (e.g., milking parlors) on such farms.

A common role of the various environmental compartments (Table 2) is the facilitation of the indirect transmission of pathogens between animals. Environmental compartments may also support a potential for extending the survival of pathogens by providing favorable conditions for their growth and multiplication [142,143]. The environmental compartments play a key role in the persistence, survival, development, and dissemination of pathogens within the farm ecosystem. Some relevant examples include the presence of trichostrongylid larvae in the soil [144], the presence of bacteria or bacteria spores on the bedding [145,146], of staphylococci on the teatcups of the milking system [147], of gastrointestinal protozoa (e.g., *Cryptosporidium* spp.) in water troughs [148], of *Sarcoptes scabiei* mites on feed troughs [149] and of *Corynebacterium pseudotuberculosis* on shears [150,151]. Hence, these compartments are not just passive surroundings, but they mediate interactions among biological components of the ecosystem.

### 5.2. Soil and Bedding

The characteristics and condition of soil on the farm grounds and the grazing paddocks can contribute to disorders of the hooves of sheep or/and goats [86,152]. The time that livestock spend grazing, coupled with increased precipitation at the location of the farm (which renders soil wet and contributes to devitalization of interdigital skin, occurring under prolonged exposure of feet to wet conditions [153,154,155]) can increase the risk of hoof disorders and consequently lameness [152,156,157].

The contamination of the soil of communal grazing paddocks with livestock excreta from other farms can be a means for the introduction of pathogens or strains of pathogens into the ecosystem, as sheep or/and goats take up these microorganisms during grazing.

Bedding within animal houses fulfills several roles in the farm ecosystem. First, it creates the physical environment in which housed animals rest; it provides thermal comfort and cleanliness and is associated with the welfare of the animals, but it also deteriorates progressively due to the accumulation of organic material [158,159]. Bedding can also be considered an interface between livestock and the waste component of the ecosystem; when used bedding is removed or applied to outside land.

### 5.3. Farm Buildings and Systems

Animal buildings and sheds are important for the housing of livestock and the provision of feed therein. However, it was reported that buildings with poor ventilation predisposed animals to increased incidence of respiratory infections, which could be further aggravated when weather conditions favored increased relative humidity within the animal barns [160,161].

The milking parlor is the most important farm system in dairy small ruminant farms [162], with various types of milking parlors and systems available for use in sheep or/and goat farms. Recent evidence has also indicated that the milking procedure is particularly stressful, with adverse effects on the welfare of livestock [163]. Machine milking leads to a reduction in the incidence of mastitis and improved quality of bulk-tank milk produced in dairy small ruminant farms [162,164], but, nevertheless, poorly maintained equipment or incorrect milking settings can adversely affect teat condition, can predispose to mastitis, and may lead to inferior milk quality [162,165].

The use of fences, which is another important farm system, contributes to increased biosecurity measures on the farm. In general, fences are used more often in farms managed under the intensive or semi-intensive system and less frequently in farms managed under extensive systems, in which animals spend substantial time grazing. Use of fences around the farm premises contributes to the reduction of incursions into the farm premises, which contributes to the reduction of, first, pathogen dissemination into the farm ecosystem and, second, livestock predation [65,166]. Fences can, nevertheless, facilitate livestock accidents due to entanglements (e.g., injuries, fractures) [167]. Recent evidence has indicated that fences may potentially have some adverse ecological effects, among them landscape fragmentation and plant diversity reduction as a consequence of uneven grazing [65,168,169].

Farm infrastructure (buildings and systems) is developed purposely by people and represents an important investment for farmers; its extent depends on the production type (e.g., no milking parlor is necessary in meat production type farms) and management system of the farm. The continued function of infrastructure depends on economic inputs and maintenance. This need for financial input for the construction and maintenance of infrastructure and systems is a distinct difference from other abiotic compartments of the ecosystem.

### 5.4. Feed and Water

#### 5.4.1. Feed

Nutrition influences animal growth and development, reproductive performance and the quantity and quality of products from small ruminants; it is involved in various physiological and health-related processes, for example immune function and development of metabolic disorders [170,171,172,173]. The type, composition and quantity of feed provided to livestock on a farm depend on a large number of variables, among them the age, breed and production stage of animals, the management system and production targets of farms, as well as the availability of grazing and feed ingredients. In general, small ruminant diet can contain roughage (through grazing or provided as harvested forage) and various amounts of concentrate feeds; the precise ratio between these two types of feeds varies in accordance with the above.

Concentrate feed can be purchased directly as ready-made or, alternatively, it can be prepared on the farm using bought-in ingredients. In some cases, farmers may also own relevant farms (e.g., maize fields) or may purchase by-products of other factories (e.g., cottonseed cake) that help to reduce feeding costs. Feeds refer to a major pathway through which resources enter the farm ecosystem.

Additionally, feed also represents an important pathway for the flow of nutrients through the farm ecosystem. For example, nitrogen enters the ecosystem through purchased feeds or vegetation consumed and is incorporated into the livestock component. Thereafter, a proportion of nitrogen is subsequently exported from the ecosystem in animal products, principally milk and meat, whereas another proportion is excreted in feces and urine. Nitrogen in excreta may enter manure or be deposited directly onto grazing land and thus become available subsequently within the soil–vegetation system, which contributes to nutrient cycling within the farm ecosystem. Health-management decisions can modify the magnitude and the direction of these flows; for example, changes in the quantity and protein content of feed provided to livestock will modify nitrogen intake, subsequently its utilization for animal production and, finally, the amount returned to the environment through excreta.

#### 5.4.2. Water

Availability of water represents an important environmental constraint in small ruminant farming, particularly in semi-extensive and extensive management systems in arid and semi-arid areas. This can depend on the availability and yield of local water sources, seasonal precipitation and drought conditions, as well as the competition for water resources with other agricultural or human uses. Water availability may influence the location of farms and grazing areas, the duration and distribution of grazing and, ultimately, the management of livestock and the farm ecosystem. Periods of reduced water availability may therefore modify interactions within the ecosystem and management decisions and considerations applied therein [147].

Thereafter, the supply of an adequate quantity of good quality water to sheep or/and goats is important for achieving high production and ensuring their optimum health. Animals should have ad libitum access to an adequate quantity of fresh water throughout the day, which also covers the relevant welfare requirements [147,174]. In sheep or/and goat farms, water can be provided through boreholes or through the local water network. In the former case, regular water testing is necessary in order to ensure that water is free of chemical contaminants or excessive mineral concentrations that may increase the risk of clinical or subclinical disorders [147].

Conversely, farms may themselves influence the quality of surrounding water resources. Runoff or leaching from areas where livestock are housed or grazed, or where manure is stored or applied, may transfer nutrients, microorganisms or residues of veterinary medicinal products to surface water or groundwater [175,176]. Additionally, the disposal of livestock carcasses into streams represents a direct flow of material from the core livestock component across the farm boundary and into the surrounding environment [177]. Thus, water also represents a pathway through which materials originating within the farm ecosystem may cross its functional boundaries and affect the wider environment.

### 5.5. Climate Variables

Climatic variables can affect livestock health and welfare and may modify the environmental conditions during housing and grazing. In farms managed under the intensive system, buildings and their insulation can buffer livestock against external climatic conditions (e.g., high temperatures) better than in extensively managed farms; in latter farms, as discussed previously, there is minimal infrastructure, which allows greater exposure of livestock to environmental variables.

Previous studies from various parts of the world reported that local breeds of small ruminants appeared to have better tolerance to local climate conditions (including high temperature) than imported breeds (e.g., Brazil [178], Ethiopia [179], India [180]). It has been hypothesized that there might exist genetic differences to heat stress adaptation and tolerance to high temperatures [181,182]. This could be mediated through a complex network of genes [183,184]. Such an adaptation may contribute to the predominance of local breeds or their crosses in farms managed under semi-extensive, extensive or very extensive systems, wherein animals are more directly exposed to prevailing climatic conditions.

Precipitation acts as an important determinant for vegetation, specifically its type, distribution and productivity [185,186]. The total amount and seasonal timing of precipitation at the farm location determine the vegetation on the farm premises and on the grazing paddocks; this is important for the coverage of nutritional needs of the farm livestock. Indeed, water is a principal resource for the growth of plants: it is necessary for cellular turgor, nutrient transport, and photosynthesis [185].

Wind and solar radiation can also influence the thermal load experienced by grazing livestock and can also modify the effective environmental temperature. Both variables can shift the effective temperature for livestock beyond that recorded by a thermometer outdoors [187].

It must be noted that climate variables are fundamentally different from the other abiotic environmental compartments, in that they cannot be controlled at farm scale, although exposure to them can be modified. Farmers can modify livestock exposure to these variables through the appropriate health management of the farm.

In the long term, changes in climate variables at the locations of farms can have direct effects on livestock health and welfare, as well as on production outcomes. These changes will require appropriate modifications in the health management applied on farms, for example changes in buildings and housing conditions, modifications in grazing and nutritional patterns, adaptations in breed selection and alterations in the timing of performing various interventions within stages of the annual production cycle. This makes the climate variables a dynamic abiotic compartment, through which an external driver, climate change, contributes to the changes performed in several interactions within the farm ecosystem.

### 5.6. Farm Waste

Waste from small ruminant farms includes livestock and other animal excreta (feces and urine), used bedding, wasted feed, wastewater from cleaning animal houses and farm systems, milk unsuitable for sale, reproductive material (e.g., placentae or aborted embryos) and animal carcasses. Farm waste can also contain various consumables associated with farm management, for example, feed bags, baling twine, gloves, containers from veterinary pharmaceutical products, etc., which have an ecologically different role than other farm waste.

Feed and water enter into the farm ecosystem for livestock to use them for covering their maintenance and production needs, and then a substantial proportion returns to the environment as excreta. Another proportion of excreta with used bedding produces manure, which may be applied onto the grazing paddocks or other agricultural land, and can improve vegetation on the land, which in turn may contribute, in the future, to meeting nutrient requirements of livestock. Material generated as farm waste can be re-used in resource cycling and contribute to the function of the farm ecosystem.

Farm waste can interact with some biotic components of the ecosystem. It can contain pathogens, as well as various non-pathogenic microorganisms. Organic waste specifically provides a substrate for dung beetles and decomposing microorganisms. Among decomposing bacteria, the main groups include Bacillota (Firmicutes), Bacteroidota (Bacteroidetes) and Pseudomonadota (Proteobacteria); all these participate in the decomposition of organic matter in sheep excreta [188,189,190].

**Figure 3:**
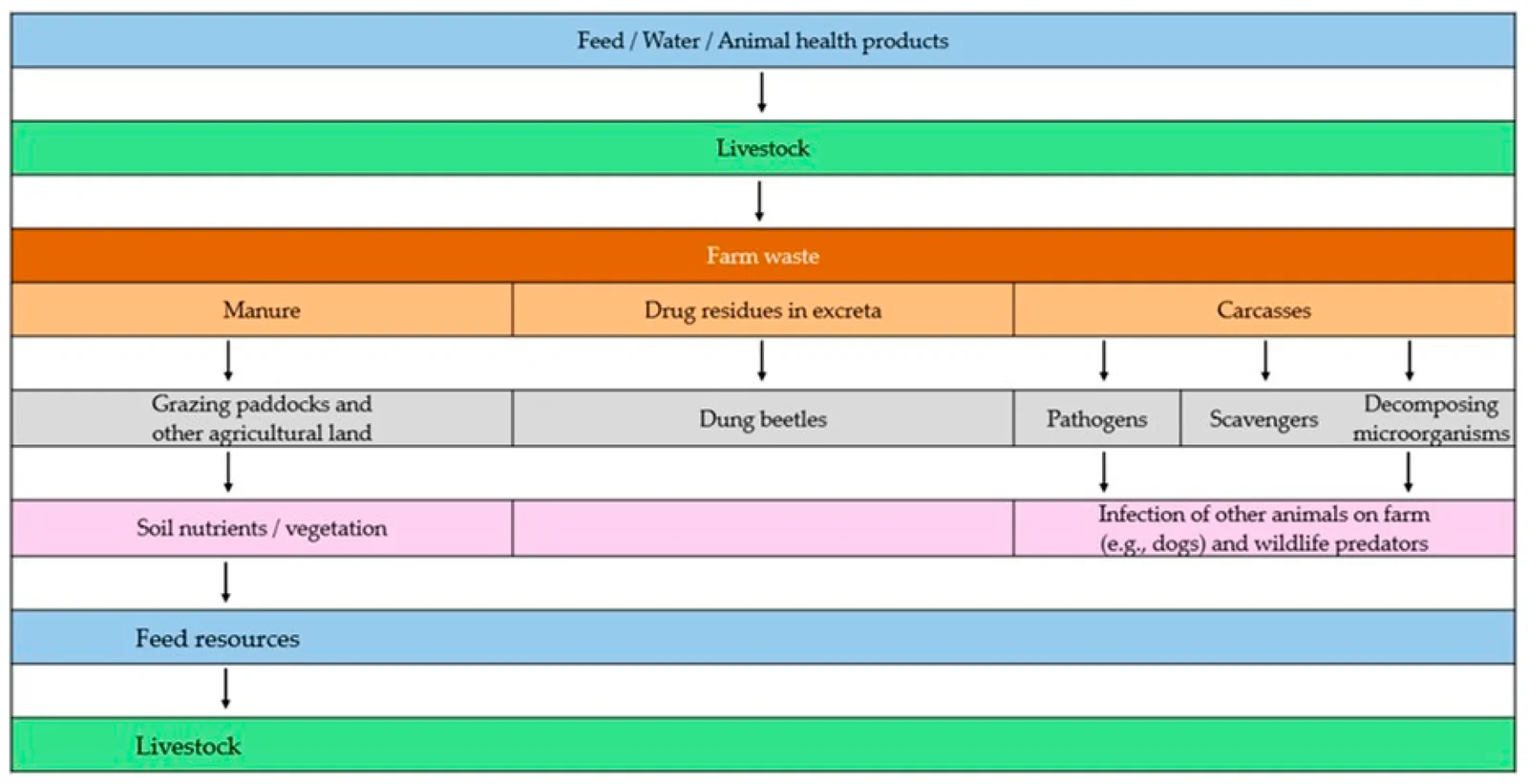
Diagram of farm-waste flows and associated interactions within small ruminant farm ecosystems.

A schematic description of farm waste flows and associated interactions is in Figure 3.

## 6. The Role of Health Management

### 6.1. General Considerations

During the establishment of the European College of Small Ruminant Health Management, small ruminant health management was defined as the evidence-based, health-oriented sheep or/and goat production management at flock or/and herd level, that gives special consideration to farm health, production systems and targets, and the management of sheep or/and goat populations. Moreover, health management includes, at animal and farm level, the evidence-based control of small ruminant diseases and disorders that adversely affect the welfare of animals and the quality and safety of products of ovine or/and caprine origin, as well as of conditions that pose a risk to public health [191]. Health management extends beyond the prevention and treatment of disease and encompasses management decisions that influence animal health, welfare, production and product quality and safety.

The farm ecosystem comprises biotic components and abiotic environmental compartments, which are connected through various and multiple interactions, as described in the previous sections. General examples of these include grazing, feeding, predation, competition, animal–human contact and their potential associations with animal health, welfare and productivity. Health management can act upon the components of the farm ecosystem and the interactions between them; it is a principal means through which humans (who themselves are a component of the ecosystem) can act upon and deliberately modify the ecosystem.

In all cases, the health management applied on farms is based on intended human decisions, which deliberately influence and direct the interactions.

### 6.2. Ecosystem-Scale Examples

**Figure 4:**
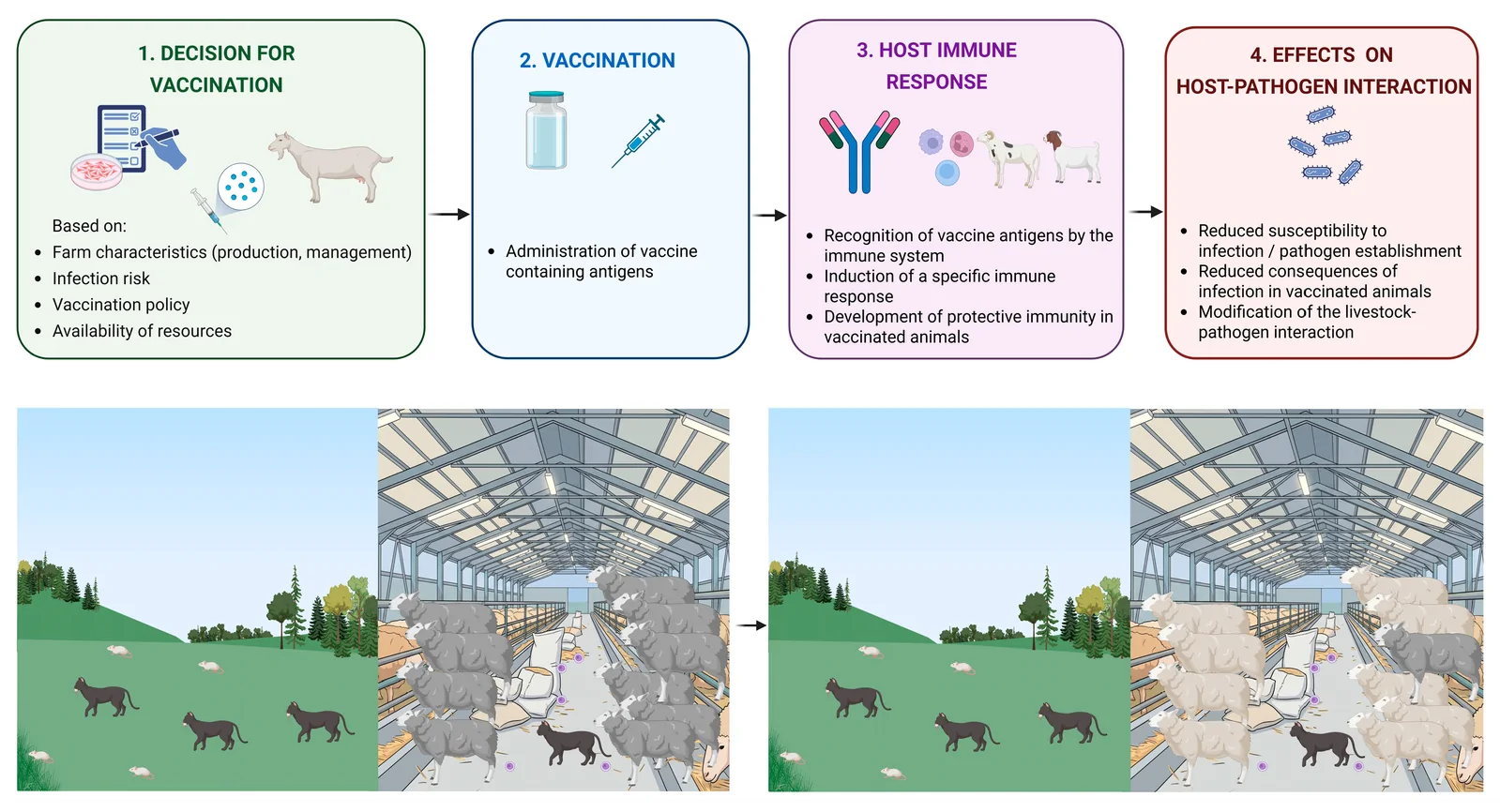
Graphical model for the role of vaccination in small ruminant farm ecosystems (figure produced in BioRender, under academic publication license no. EJ2A8PNI31 (2026)) (dark grey sheep: unvaccinated, with no vaccine-induced immunity; off-white sheep: vaccinated, with vaccine-induced immunity; purple round shapes: *T. gondii* oocysts).

**Figure 5:**
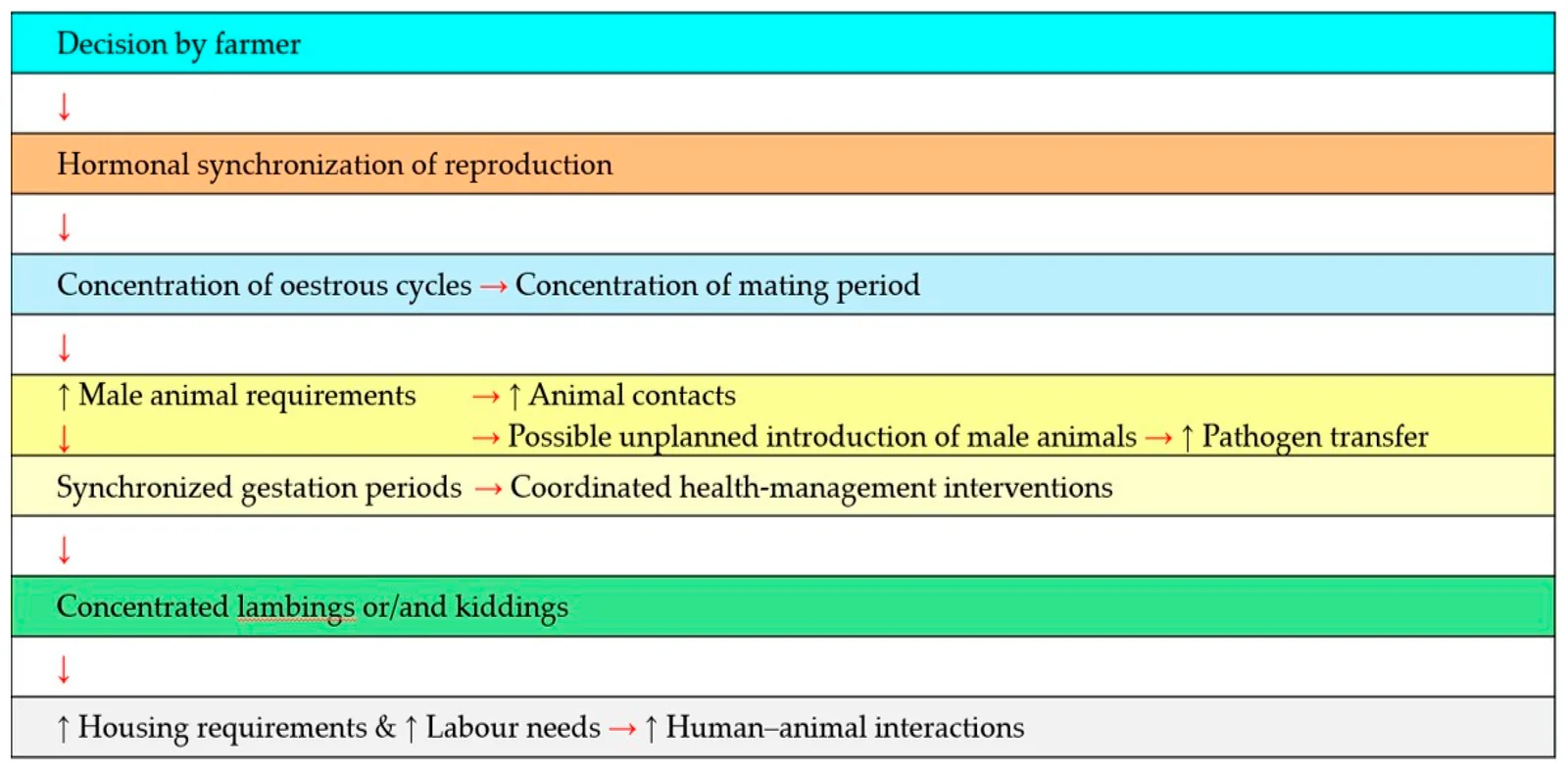
Diagram of the role of hormonal control of reproduction and subsequent interactions within small ruminant farm ecosystems (red arrows indicate flows; black arrows indicate changes in magnitude).

- Various biosecurity measures can be implemented based on decisions of the farmer and lead to setting up fencing of the farm premises, controlling access to the farm or/and quarantining newly purchased animals, which all lead to modifying contact patterns between livestock and people (e.g., visitors), other animals (e.g., predator mammals) and pathogens (e.g., bacterial transfer).
- Vaccinations are applied based on decisions of the farmer and provide a biological input into livestock (antigens). This induces or enhances specific immunity in the sheep or/and goat population, and modifies the interactions between vaccinated livestock and the targeted pathogen population (e.g., *Toxoplasma gondii*) and the potential consequences of livestock infection (Figure 4). The application of vaccination as a health-management intervention is also dependent on the availability of an appropriate vaccine against the pathogen concerned; vaccine availability can thus act as an external driver influencing health-management decisions at farm level.
- The application of hormonal control of reproduction, using intravaginal insertion of progestogens and injectable administration of equine chorionic gonadotrophin leads to concentration of the mating period within a short period (5–7 days) and requires an increased number of rams or/and bucks for improving conception rates, which increases direct contact of animals and consequently dissemination of pathogens during the mating season. If these animals are not available on a farm, they could be transferred from other farms, which may reduce biosecurity standards applied and create biosecurity challenges, potentially resulting in the introduction of new pathogens into the farm. The synchronization of estrous cycles of the female animals leads to their gestation periods running almost simultaneously, which supports grouping pregnant females for the concurrent administration of vaccines and preventive treatments towards the final stage of pregnancy. Later, the majority of females give birth within a short period (10–14 days), which requires availability of infrastructure to set up pens for the post-parturient animals and their offspring, as well as increased farm labor to monitor animals and provide assistance to newborns. A schematic description of this example is in Figure 5.
- An increase in the number of daily milking sessions in a dairy farm is carried out based on decisions of the farmer and leads to increased use of the milking system, handling of livestock and labor requirements, which modify livestock–equipment and livestock–human interactions and also require increased water use, with effects on animal welfare, udder health and milk yield, as well as with larger amount of wastewater; also, the increased number of livestock passages through the milking parlour contributes to increased contact among animals, as well as with the parlour equipment (e.g., teatcups), leading to higher opportunities for pathogen circulation. In this example, it should again be noted that the production of increased volumes of wastewater and the higher pathogen circulation were unintended effects of the health management applied on this occasion.
- Nitrogen enters into the farm ecosystem principally through feeds, as mentioned above, as well as through animals introduced into the farm (e.g., replacement ewes or does). Part of the nitrogen taken up by livestock is exported from the ecosystem through milk and meat and through animals sold from the farm, whilst other parts return to the environment through excreta or enters the farm-waste stream through animal carcasses. In cases of mastitis, there is a documented decrease in the total milk protein produced; further, lambs or/and kids may also be affected, showing suboptimal growth and potentially reduced protein accretion. Moreover, acute clinical mastitis may result in animal death; increased replacement rates may also be required to compensate for the increased mortality and culling rates associated with the infection, leading to additional livestock inputs into the ecosystem. In this way, mastitis can modify the partitioning and flows of nitrogen through the livestock component, as well as the proportions of nitrogen exported in animal products or transferred into farm waste. Udder-health management measures applied for the control of mastitis (e.g., milking-parlor hygiene, vaccination, intramammary antibiotic administration at the end of the lactation period), through maintaining mammary health and production, can therefore indirectly influence these material flows within the farm ecosystem.
- Anthelmintic treatments are performed based on decisions of the farmer and act upon the livestock and the endoparasites (e.g., gastrointestinal trichostrongylids), which leads to improved animal health and higher production outputs, and, thereafter, to the presence of drug residues in excreta and in farm waste; this can affect the viability of dung beetles in the soil of farm premises and grazing paddocks. Moreover, anthelmintic treatment also imposes selection pressure on parasite populations, favoring the survival and reproduction of resistant parasites and, consequently, potentially increasing the frequency of resistance within the parasite population. This effect can accumulate after repeated treatments and can be accentuated in cases of erroneous decisions (e.g., in cases of administration of a reduced dose of the anthelmintic, because of, for example, underestimation of the bodyweight of animals), thereby representing a management-induced modification of a biotic component of the farm ecosystem, which extends beyond the immediately intended outcome of treatment. Maintenance of parasite populations in refugia, which remain unexposed to treatment, can reduce selection pressure by preserving susceptible parasites within the ecosystem and represents an example of health management deliberately modifying the structure and interactions of a parasite population. Additionally, residues of administered anthelmintics may be excreted in feces and enter farm waste and soil, where they can affect non-target organisms such as dung beetles. Thus, the same health-management intervention can simultaneously modify the targeted parasite population through selection and non-target components through pharmaceutical residues.
- Administration of antibiotics provides a further example of selection pressure generated through farm health management. Their administration increases the risk of selection of resistant bacterial populations within the treated livestock, whilst antibiotic residues, resistant bacteria, and also resistance genes may subsequently enter into farm waste through feces, urine, or other biological material. Through manure, used bedding or wastewater, these may reach soil and other environmental compartments and potentially circulate among microbial communities within or beyond the farm ecosystem. Also, cell-free genetic material derived from antimicrobial-resistant bacteria in milk can persist during processing [192,193], and become available for subsequent dissemination. Antimicrobial resistance therefore illustrates how a health-management intervention directed at the livestock component may modify microbial biotic components and generate flows through several abiotic environmental compartments of the farm. In this last case, milk produced on a farm and derived dairy products may provide a route for the transfer of these genes from the farm ecosystem to the human population.
- Another directional flow influenced by health management refers to the use of veterinary medicines and the subsequent production of food from sheep or goats. Following administration of a veterinary medicine to livestock, residues of the active substance or its metabolites may occur in edible products. Observance of the prescribed withdrawal period (defined as the minimum amount of time that must elapse between the final administration of a veterinary medicine to an animal and the time when products from the treated animal can be safely supplied for human consumption) represents a human management decision, which determines when these products can leave the farm ecosystem and enter into the food chain. Thus, health management can regulate a directional flow extending from the input of a pharmaceutical product, through the livestock component and animal products, to consumers outside the farm ecosystem.

### 6.3. Integrated Application of Health Management in the Ecosystem Framework

Considered together, the above examples highlight that health-management interventions can modify multiple components and compartments and interactions and flows within the farm ecosystem. The effects may also modify the temporal organization of the system (e.g., as in the case of application of hormonal control of reproduction), as well as extend beyond their immediately intended targets.

**Figure 6:**
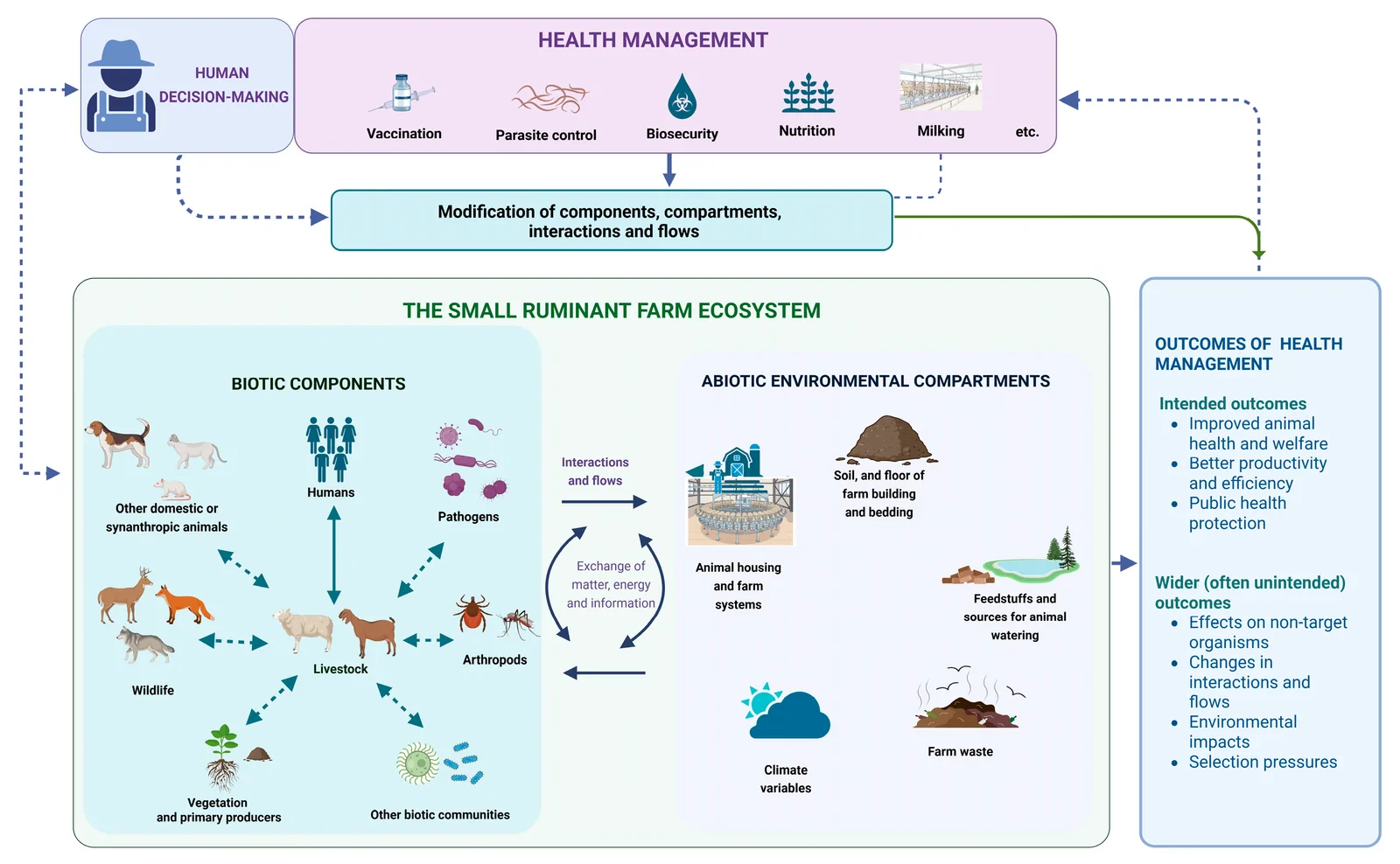
Graphical model for the framework of the small ruminant farm as a managed ecosystem and its modifications by means of health management (figure produced in BioRender, under academic publication license no. AF2A8PO67O (2026)) (arrows indicate representative interactions).

This also places humans in a dual position within the ecosystem, first as agents able to modify the ecosystem and the interactions taking place through farm management decisions, including those related to the application of health management, and second, as a biotic component of the ecosystem (Figure 6).

## 7. Conclusions

### 7.1. Conceptual Contribution of the Proposed Framework

We propose a reframing of the current concept, in which small ruminant farms are considered as managed ecosystems. Moreover, we present and characterize the principal biotic components and abiotic environmental compartments of the farm ecosystem within this reframed concept, and we also describe various interactions that could occur within and between these components. We propose that the ecosystem of a farm can extend beyond the physical boundaries of that farm.

Our paradigm moves the conceptualization of small ruminant health and production beyond approaches that consider individual components or interactions in isolation. The proposed framework conceptualizes small ruminant farms as managed ecosystems, in which biotic components and abiotic environmental compartments are interconnected through dynamic interactions and flows modified by human management and external drivers. This novel conception integrates the ecosystem components and compartments within a single framework that allows and supports the systematic consideration of their relationships, directionality, boundaries, and management-mediated modifications. The intended contribution does not refer to the identification of individual components/compartments or interactions, many of which are already well recognized, but to their integration within a single framework; through this, these dimensions can be examined systematically within the functioning of an individual small ruminant farm ecosystem.

Health management is a human-directed process acting upon the ecosystem. Health management and the interventions and tools that it encompasses can modify relationships and interactions among components and compartments of the farm ecosystem. The objective of these interventions is to achieve optimum health and welfare for the animals, at the same time maximizing production outcomes and safeguarding public health. The resulting effects of these interventions may extend beyond their immediate intended targets. In this model, the dual role of humans should also be noted.

The proposed framework is distinct from, but complementary to, existing approaches used in livestock studies. One Health emphasizes the interdependence of animal, human, and environmental health; agroecosystem approaches consider agricultural production within broader ecological processes; and socio-ecological approaches emphasize interactions between humans and ecological systems. Herd health approaches, in turn, focus principally on maintaining and improving health and productivity at the population level through coordinated management. The framework proposed herein draws on elements of those perspectives, but considers the individual sheep or/and goat farm as the principal unit of analysis and explicitly organizes its biotic components and abiotic environmental compartments according to the interactions and flows connecting them, their directionality, inputs and outputs across functional boundaries, and human management and feedback.

### 7.2. Future Perspectives and Applications

The multiplicity of components, environmental compartments, interactions, flows and management interventions within the farm ecosystem also highlights the value of Precision Livestock Farming approaches in small ruminant health management [194,195,196]. Integration of data describing these different dimensions can facilitate the application of machine-learning methods to identify complex relationships, predict health and production outcomes, and support management decisions at farm level [197].

The framework proposed in this paper provides the basis for the further development of health management of small ruminant farms as managed ecosystems. The consideration of these wider relationships can support better-informed and integrated health management of sheep or/and goat farms and their animal populations.

As a qualitative conceptual framework, it provides a basis for the future quantitative characterization of individual interactions and flows. In this context, future work can quantify the effects of the various interactions and flows within farm ecosystems; it can also evaluate how their structure and relative importance differ among production types, management systems and geographical settings. Future studies can use network-based approaches to identify components or interactions with disproportionate influence on small ruminant health and welfare and production outcomes. The complexity of these relationships highlights the importance of interdisciplinary work in these studies.

## Figures and Tables

**Table 1:** Biotic components of the ecosystem in small ruminant farms and examples of their content.

Biotic Components	Examples of Content
Livestock	Sheep or/and goats
Humans	People working on farms: farmer and family, farm workers
	People functionally associated with farms: veterinarians, technical personnel
	People temporarily interacting with farms: hunters, occasional visitors
Other domestic or synanthropic animals on farms	Dogs, other farm animals (cattle, camels, pigs, rabbits), poultry, equines, cats, rodents
Wildlife	Wildlife ruminants, predatory mammals, wild rodents, reptiles, birds flying over farms
Vegetation and primary producers	Pasture vegetation, rangeland shrubs, forage crops, weeds, toxic plants
Pathogens	Prions, viruses, bacteria, fungi, protozoa, metazoan endoparasites, ectoparasites
Arthropods	Ticks, midges, mosquitoes, flies, dung beetles
Other biotic communities	Livestock-associated microbiota: rumen, skin and udder microbiomes, methanogenic Archaea
	Feed-associated microbiota: lactic acid bacteria in silage
	Soil biota: mycorrhizal fungi, free-living nematodes, earthworms

Underlined text indicates subcategories of the same listed biotic component.

**Table 2:** Examples of abiotic environmental compartments for small ruminant farms.

Abiotic Environmental Compartments	Examples of Content
Soil	(potentially contaminated with animal excreta)
Animal housing and associated areas and materials	Constructions and areas: barns and sheds, yards
	Floors and bedding: concrete or earth floors, bedding materials
Farm systems	fencing material, milking system, milk-replacer preparation and administration system, housing stalls, feed or water troughs
Machinery	tractors, feed wagons, vehicles
Farm light equipment	shears, animal-health-related equipment
Feedstuffs	concentrated feeds, hay, silage
Sources for animal watering	water troughs, streams
Climate variables	temperature, precipitation, wind
Farm waste	manure, livestock carcasses

Underlined text indicates subcategories of the same listed abiotic environmental compartment.

**Table 3:** Summary presentation of the elements of small ruminant farm ecosystems with relevant representative examples of interactions.

Principal Interaction/Flow	Components/ Compartments Involved ^1^	Directionality	Inputs/Outputs	Boundaries	Human Management and Feedback	Principal Moderating Variables
**Grazing**	Livestock ↔ vegetation, soil	Bidirectional/ cyclical	Nutrient input to livestock, excreta returned	May extend beyond farm premises	Grazing allocation, stocking-rate decisions	Management system, geography, season
**Predation**	Wildlife → livestock	Predominantly unidirectional	Wildlife incursion, livestock loss	Crosses physical boundaries	Fencing, guardian animals	Geography, management system
**Livestock–wildlife pathogen transmission**	Livestock ↔ wildlife, shared environment	Uni- or bidirectional	Pathogen introduction/dissemination	Frequently crosses/overlaps physical and functional boundaries	Biosecurity, grazing management	Geography, wildlife populations, management system
**Livestock–human contact**	Humans ↔ livestock	Bidirectional interaction	Human entry/exit, products/information	Crosses physical and functional boundaries	Handling, veterinary interventions, hygiene	Production type, labour, management system
**Manure/nutrient cycling**	Livestock → waste → soil → vegetation → livestock	Cyclical	Excreta/manure, nutrients recycled or exported	Internal or crosses boundaries if manure is removed or applied elsewhere	Waste/manure management	Housing/grazing system, geography
**Feed purchase and use**	External environment → feed → livestock	Predominantly unidirectional input	Feed/nutrient input, products/excreta downstream	Crosses physical and functional boundaries	Nutritional management	Production type, management system, economics
**Animal movements**	External livestock population → farm livestock	Predominantly unidirectional per movement	Animals/pathogens/genetic material	Explicit boundary crossing	Replacement policy, quarantine/biosecurity	Production type, management system, geography
**Communal grazing/transhumance**	Livestock from multiple farms, wildlife, vegetation, soil, water	Multiple/bidirectional	Livestock and associated biological/material ^2^ flows moving between locations	Seasonally dynamic, overlapping with those of other systems	Multiple farmers, coordination potentially required	Extensive/transhumant management, geography, season

^1^ ↔ indicates bidirectional flow, → indicates unidirectional flow; ^2^ For example, nutrients or pathogens.

## Data Availability

No new data were created or analyzed in this study. Data sharing is not applicable to this article. All data discussed are contained within the article and its Supplementary Materials.

## Supplementary Materials

The following supporting information can be downloaded at: https://www.mdpi.com/article/10.3390/ani16193067/s1, Table S1: Management systems applied in sheep farms and the principal elements of these systems (source: reproduced from the EFSA Panel on Animal Health and Welfare); Table S2: Examples of wildlife mammal predators for sheep or/and goats that predominate in various regions of the world; Table S3: Examples of pathogens that infect sheep or/and goats.

## References

[1] Chapin F.S., Matson P.A., Vitousek P.M. (2011). Principles of Terrestrial Ecosystem Ecology.

[2] Tansley A.G. (1935). The use and abuse of vegetational concepts and terms. Ecology.

[3] Odum E.P. (1971). Fundamentals of Ecology.

[4] Willis A.J. (1977). The ecosystem: An evolving concept viewed historically. Funct. Ecol..

[5] Swift M.J., Vandermeer J., Ramakrishnan P.S., Anderson J.M., Ong C.K., Hawkins B.A., Mooney H.A., Cushman J.H., Medina F., Sala O.E., Schulze E.D. (1996). Biodiversity and agroecosystem function. Functional Roles of Biodiversity: A Global Perspective.

[6] Gliessman S. (2015). Agroecology: A growing field. Agroecol. Sustain. Food Syst..

[7] Tilman D., Cassman K.G., Matson P.A., Naylor R., Polasky S. (2002). Agricultural sustainability and intensive production practices. Nature.

[8] EFSA AHAW Panel (EFSA Panel on Animal Health and Welfare) (2014). Scientific opinion on the welfare risks related to the farming of sheep for wool, meat and milk production. EFSA J..

[9] Kilgour R.J., Waterhouse T., Dwyer C.M., Ivanov I.D., Dwyer C.M. (2008). Farming systems for sheep production and their effect on welfare. The Welfare of Sheep.

[10] Vaitsi G.A., Bourganou M.V., Michael C.K., Vasileiou N.G.C., Katsarou E.I., Katsafadou A.I., Gougoulis D.A., Mavrogianni V.S., Fthenakis G.C. (2026). Mapping the scientific literature on sheep and goat research: General appraisal and significance of the year of publication. Animals.

[11] Salaheen S., Chowdhury N., Hanning I., Biswas D. (2015). Zoonotic bacterial pathogens and mixed crop-livestock farming. Poult. Sci..

[12] Jori F., Hernandez-Jover M., Magouras I., Dürr S., Brookes V.J. (2021). Wildlife-livestock interactions in animal production systems: What are the biosecurity and health implications?. Anim. Front..

[13] Zhang T., Nickerson E., Zhang W., Peng X., Shang Y., Zhou Y., Luo Q., Wen G., Cheng Z. (2024). The impacts of animal agriculture on One Health—Bacterial zoonosis, antimicrobial resistance, and beyond. One Health.

[14] Dougherty H.C., Ahmadi A., Oltjen J.W., Mitloehner F.M., Kebreab E. (2019). Modeling production and environmental impacts of small ruminants—Incorporation of existing ruminant modeling techniques, and future directions for research and extension. Appl. Anim. Sci..

[15] Kannan G., Kannan G. (2025). Welfare issues related to small ruminant production and sustainability: An overview. Small Ruminant Welfare, Production and Sustainability.

[16] Kandziora M., Burkhard B., Müller F. (2013). Interactions of ecosystem properties, ecosystem integrity and ecosystem service indicators—A theoretical matrix exercise. Ecol. Indic..

[17] Zaman W., Ayaz A., Puppe D. (2025). Biogeochemical cycles in plant-soil systems: Significance for agriculture, interconnections, and anthropogenic disruptions. Biology.

[18] Strayer D.L., Power M.E., Fagan W.F., Pickett S.T.A., Belnap J. (2003). A classification of ecological boundaries. BioScience.

[19] Post D.M., Doyle M.W., Sabo J.L., Finlay J.C. (2007). The problem of boundaries in defining ecosystems: A potential landmine for uniting geomorphology and ecology. Geomorphology.

[20] Geddes D. (1982). Neolithic transhumance in the Mediterranean Pyrenees. World Archaeol..

[21] McGuirk E.F., Nunn N. (2025). Transhumant pastoralism, climate change, and conflict in Africa. Rev. Econ. Stud..

[22] Pulina G., Milán M.J., Lavín M.P., Theodoridis A., Morin E., Capote J., Thomas D.L., Francesconi A.H.D., Caja G. (2018). Current production trends, farm structures, and economics of the dairy sheep and goat sectors. J. Dairy Sci..

[23] Mateo J., Caro I., Carballo D.E., Gutiérrez-Méndez N., Arranz J.J., Gutiérrez-Gil B. (2018). Carcass and meat quality characteristics of Churra and Assaf suckling lambs. Animal.

[24] Lagonikou M., Tsimpouri E., Gelasakis D.E., Denezi E., Gelasakis A.I. (2024). Prediction of carcass traits in fattening Chios and Serres lambs using real-time ultrasonography and live body weight measurements pre-slaughter. Meat Sci..

[25] European Commission (2023). Agriculture and Rural Development; Young Farmers. https://agriculture.ec.europa.eu/common-agricultural-policy/income-support/young-farmers_en#young-farmer-definition.

[26] Papadopoulos A.G., Fratsea L.M., Spyrellis S., Baltas P. (2021). Exploring the contribution of migrant labour in Greek agriculture. It. Rev. Agric. Econ..

[27] Payan T., Rodríguez-Sánchez J.I. (2024). Migrant Workers’ Vital Role in Agriculture: A Conversation with Alejandro Gutiérrez-Li. Rice University’s Baker Institute for Public Policy. https://www.bakerinstitute.org/research/migrant-workers-vital-role-agriculture-conversation-alejandro-gutierrez-li.

[28] Davis B., Mane E., Gurbuzer L.Y., Caivano G., Piedrahita N., Schneider K., Azhar N., Benali M., Chaudhary N., Rivera R. (2026). A new estimate for global and country-level employment in agrifood systems. World Dev..

[29] World Organisation for Animal Health Old Classification of Diseases Notifiable to the OIE—List. https://www.woah.org/en/what-we-do/animal-health-and-welfare/animal-diseases/old-classification-of-diseases-notifiable-to-the-oie-list-a/.

[30] World Organisation for Animal Health Events Management, Country/Territory: Greece, 2026. https://wahis.woah.org/#/event-management.

[31] Sampazioti G. (2026). Alert for Feta Cheese: Stocks May not Suffice Until the End of the Year, Price Increasing to 15–16 Euros. https://www.newmoney.gr/roh/palmos-oikonomias/oikonomia/sinagermos-gia-ti-feta-ta-apothemata-isos-den-vgaloun-ti-chronia-pros-ta-15-16-evro-i-timi/.

[32] Turner M.G., Calder W.J., Cumming G.S., Hughes T.P., Jentsch A., LaDeau S.L., Lenton T.M., Shuman B.N., Turetsky M.R., Ratajczak Z. (2020). Climate change, ecosystems and abrupt change: Science priorities. Philos. Trans. R. Soc. Lond. B Biol. Sci..

[33] Bishop S.C. (2015). Genetic resistance to infections in sheep. Vet. Microbiol..

[34] McDonald P., Edwards R., Greenhalgh J.F.D., Morgan C., Sinclair L., Wilkinson R. (2011). Animal Nutrition.

[35] Fthenakis G.C., Arsenos G., Brozos C., Fragkou I.A., Giadinis N.D., Giannenas I., Mavrogianni V.S., Papadopoulos E., Valasi I. (2012). Health management of ewes during pregnancy. Ani. Rep. Sci..

[36] Anuta A., Wang X., Kinay P. (2025). The impacts of climate change on livestock: An interdisciplinary, scoping review of health, production, and adaptation strategies. Clim. Smart Agric..

[37] Martínez-Ibáñez E., Laso J., Margallo M., Aldaco R. (2026). Life cycle assessment of livestock production: A systematic review of methodological advances, harmonization challenges, and future directions. Clean. Environ. Syst..

[38] Fountain J., Woodgate R., Rast L., Hernández-Jover M. (2018). Assessing biosecurity risks for the introduction and spread of diseases among commercial sheep properties in New South Wales, Australia, using foot-and-mouth disease as a case study. Front. Vet. Sci..

[39] Lee G.-J., Pak S.-I., Lee K.-N., Hong S. (2019). Movement-based biosecurity zones for control of highly infectious animal diseases: Application of community detection analysis to a livestock vehicle movement network. Sustainability.

[40] Gehring T.M., VerCauteren K.C., Landry J.M. (2010). Livestock protection dogs in the 21st century: Is an ancient tool relevant to modern conservation challenges?. BioScience.

[41] Sepúlveda M.A., Singer R.S., Silva-Rodríguez E., Stowhas P., Pelican K. (2014). Domestic dogs in rural communities around protected areas: Conservation problem or conflict solution?. PLoS ONE.

[42] Moreira-Arce D., Ugarte C.S., Zorondo-Rodríguez F., Simonetti J.A. (2018). Management tools to reduce carnivore-livestock conflicts: Current gap and future challenges. Rangel. Ecol. Manag..

[43] Katsarou E.I., Arsenopoulos K.V., Michael C.K., Lianou D.T., Petinaki E., Papadopoulos E., Fthenakis G.C. (2024). Gastrointestinal helminth infections in dogs in sheep and goat farms in Greece: Prevalence, involvement of wild canid predators and use of anthelmintics. Animals.

[44] Ivaşcu C.M., Biro A. (2020). Coexistence through the ages: The role of native livestock guardian dogs and traditional ecological knowledge as key resources in conflict mitigation between pastoralists and large carnivores in the Romanian Carpathians. J. Ethnobiol..

[45] Mugnier S., Husson C., Cournut S. (2021). Why and how farmers manage mixed cattle–sheep farming systems and cope with economic, climatic and workforce-related hazards. Renew. Agric. Food Syst..

[46] Prache S., Vazeille K., Chaya W., Sepchat B., Note P., Sallé G., Veysset P., Benoît M. (2023). Combining beef cattle and sheep in an organic system. I. Co-benefits for promoting the production of grass-fed meat and strengthening self-sufficiency. Animal.

[47] Buxton D., Martin W.B., Aitken I.D. (2000). Toxoplasmosis and neosporosis. Diseases of Sheep.

[48] Dubey J.P. (2009). Toxoplasmosis in sheep—The last 20 years. Vet. Parasitol..

[49] Benavides J., González-Warleta M., Arteche-Villasol N., Pérez V., Mezo M., Gutiérrez-Expósito D. (2022). Ovine neosporosis: The current global situation. Animals.

[50] Mendoza-Morales L.F., Lagorio V., Corigliano M.G., Sánchez-López E., Ramos-Duarte V.A., Clemente M., Sander V.A. (2022). Neosporosis in sheep: A systematic review and meta-analysis of global seroprevalence and related risk factors. Acta Trop..

[51] Farhab M., Aziz M.W., Shaukat A., Cao M.-X., Hou Z., Huang S.-Y., Li L., Yuan Y.-G. (2025). Review of toxoplasmosis: What we still need to do. Vet. Sci..

[52] Witmer G. (2022). Rodents in agriculture: A broad perspective. Agronomy.

[53] Huels F., Jacob J. (2026). Rodent-borne pathogens as economic and zoonotic health threat to livestock farming: A review. One Health.

[54] Foreyt W.J., Jenkins E.J., Appleyard G.D. (2009). Transmission of lungworms (*Muellerius capillaris*) from domestic goats to bighorn sheep on common pasture. J. Wildl. Dis..

[55] Winter J., Rehbein S., Joachim A. (2018). Transmission of helminths between species of ruminants in Austria appears more likely to occur than generally assumed. Front. Vet. Sci..

[56] Khanyari M., Oyanedel R., Khara A., Sharma M., Milner-Gulland E.J., Suryawanshi K.R., Vineer H.R., Morgan E.R. (2024). Predicting and reducing potential parasite infection between migratory livestock and resident Asiatic ibex of Pin valley. Ind. J. Biosci..

[57] Katsarou E.I., Michael C.K., Arsenopoulos K.V., Lianou D.T., Liagka D.V., Mavrogianni V.S., Papadopoulos E., Fthenakis G.C. (2025). A note on some health-related outcomes in small ruminant farms with common grazing with wildlife ruminants. Animals.

[58] Rodolakis A. (2006). Q fever, state of art: Epidemiology, diagnosis and prophylaxis. Small Rumin. Res..

[59] Berri M., Rekiki A., Boumedine K.S., Rodolakis A. (2009). Simultaneous differential detection of *Chlamydophila abortus*, *Chlamydophila pecorum* and *Coxiella burnetii* from aborted ruminant’s clinical samples using multiplex PCR. BMC Microbiol..

[60] Billinis C. (2013). Wildlife diseases that pose a risk to small ruminants and their farmers. Small Rumin. Res..

[61] Allen L.R., Sparkes E.C. (2001). The effect of dingo control on sheep and beef cattle in Queensland. J. Appl. Ecol..

[62] Chetri M., Odden M., Wegge P. (2017). Snow leopard and Himalayan wolf: Food habits and prey selection in the Central Himalayas, Nepal. PLoS ONE.

[63] Hamid A., Mahmood T., Fatima H., Hennelly L.M. (2019). Origin, ecology and human conflict of gray wolf (*Canis lupus*) in Suleman Range, South Waziristan, Pakistan. Mammalia.

[64] Nattrass N., Drouilly M., O’Riain M.J. (2020). Learning from science and history about black-backed jackals *Canis mesomelas* and their conflict with sheep farmers in South Africa. Mamm. Rev..

[65] McInturff A., Miller J.R.B., Gaynor K.M., Brashares J.S. (2021). Patterns of coyote predation on sheep in California: A socio-ecological approach to mapping risk of livestock-predator conflict. Conserv. Sci. Pract..

[66] Ballarín J., García-Serrano A., Herrero J., Reiné R. (2023). Shepherds view of large carnivore recovery in the Pyrenees, Spain. Animals.

[67] Katsarou E.I., Reid N., Lianou D.T., Fthenakis G.C. (2024). Stress related to wild canid predators near dairy sheep farms associated with increased somatic cell counts in bulk-tank milk. Sci. Rep..

[68] Torres-Romero E.J., Bender L.C. (2024). Extent and patterns of livestock depredation in Mexico. Rangel. Ecol. Manag..

[69] Brunschwig G., Pinot A., Brosse-Genevet E., Virapin M., Garde L. (2026). Predation impacts of dogs on flocks of sheep in wolf-free areas: A synthesis based on surveys conducted in 11 breeding territories in France. Small Rumin. Res..

[70] Nattrass N., Conradie B. (2018). Predators, livestock losses and poison in the South African Karoo. J. Clean. Prod..

[71] Shibru S., Tolcha A., Melese M., Ayechew B., Tsegaye G., Gatew S. (2024). Perceptions of farmers towards carnivores in an Ethiopian protected area. Afr. J. Ecol..

[72] Bleicher S.S. (2017). The landscape of fear conceptual framework: Definition and review of current applications and misuses. Peer J..

[73] Laundré J.W., Hernández L., Altendorf K.B. (2001). Wolves, elk, and bison: Reestablishing the “landscape of fear” in Yellowstone National Park, USA. Can. J. Zool..

[74] Laundré J.W., Hernandez L., Ripple W.J. (2010). The landscape of fear: Ecological implications of being afraid. Open Ecol. J..

[75] Soofi M., Soufi M., Royle A., Waltert M., Khorozyan I. (2022). Numbers and presence of guarding dogs affect wolf and leopard predation on livestock in northeastern Iran. Bas. Appl. Ecol..

[76] Smith B.R., Whitehouse-Tedd K., Yarnell R.W., Marginean M., Popa R., Morley A., Trewby I., Uzal A. (2025). Direct interactions between livestock guarding dogs and wildlife in a transhumance grazing system. Conserv. Sci. Pract..

[77] García M.L.R., Lina M., Martínez S.D., Caruso N., Casanave E.B., Vidal E.M.L., Lucherini M. (2025). Pumas and livestock guarding dog interactions: A case study from an area of human-carnivore conflict in central Argentina. Oryx.

[78] Owen M.R., Trees A.J. (1998). Vertical transmission of *Toxoplasma gondii* from chronically infected house (*Mus musculus*) and field (*Apodemus sylvaticus*) mice determined by polymerase chain reaction. Parasitology.

[79] Sumiyama D., Izumiya H., Kanazawa T., Murata K. (2014). *Salmonella* infection in green anoles (*Anolis carolinensis*), an invasive alien species on Chichi Island of the Ogasawara archipelago in Japan. J. Vet. Med. Sci..

[80] Chatzopoulos D.C., Sarrou S., Vasileiou N.G.V., Ioannidi K.S., Peteinaki E., Valiakos G., Tsokana C.N., Papadopoulos E., Spyrou V., Mavrogianni V.S. (2016). Dissemination of intestinal pathogens between lambs and puppies in sheep farms. Small Rumin. Res..

[81] Jameson T.J.M., Johnston G.R., Barr M., Sandow D., Head J.J., Turner E.C. (2024). Squamate scavenging services: Heath goannas (*Varanus rosenbergi*) support carcass removal and may suppress agriculturally damaging blowflies. Ecol. Evol..

[82] Aklilu A.Z., Elofsson K., Halvarsson P., Kjellander P., Höglund J. (2024). A pound for information or a penny for cure: Farmers’ economic decisions on testing and treatment of livestock diseases. Aus. J. Agric. Resour. Econ..

[83] Corner-Thomas R.A., Kenyon P.R., Morris S.T., Ridler A.L., Hickson R.E., Greer A.W., Logan C.M., Blair H.T. (2015). Influence of demographic factors on the use of farm management tools by New Zealand farmers. N. Z. J. Agric. Res..

[84] Tizard I.R. (2021). Vaccines for Veterinarians.

[85] Mavrogianni V.S., Menzies P.I., Fragkou I.A., Fthenakis G.C. (2011). Principles of mastitis treatment in sheep and goats. Vet. Clin. N. Am. Food Anim. Pract..

[86] Winter A.C. (2009). Footrot control and eradication (elimination) strategies. Small Rumin. Res..

[87] Tauer L. (1995). Age and farmer productivity. Rev. Agric. Econ..

[88] Lianou D.T., Fthenakis G.C. (2022). Use of antibiotics against bacterial infections on dairy sheep and goat farms: Patterns of usage and associations with health management and human resources. Antibiotics.

[89] Provenza F.D., Scott C.B., Phy T.S., Lynch J.J. (1996). Preference of sheep for foods varying in flavors and nutrients. J. Anim. Sci..

[90] Ginane C., Baumont R., Favreau-Peigné A. (2011). Perception and hedonic value of basic tastes in domestic ruminants. Physiol. Behav..

[91] Albanell E., Manuelian C.L., Rovai M., Salama A.A.K., Caja G. (2017). Using long-term averted goats for selective grazing in olive groves. Animal.

[92] Fthenakis G.C., Manos G., Brozos C. (2001). Plants and phytotoxins causing toxic effects in embryos of ewes or does. J. Hell. Vet. Med. Soc..

[93] Athanasiadou S., Kyriazakis I., Jackson F., Coop R.L. (2001). Direct anthelmintic effects of condensed tannins towards different gastrointestinal nematodes of sheep: In vitro and in vivo studies. Vet. Parasitol..

[94] Iqbal Z., Sarwar M., Jabbar A., Ahmed S., Nisa M., Sajid M.S., Khan M.N., Mufti K.A., Yaseen M. (2007). Direct and indirect anthelmintic effects of condensed tannins in sheep. Vet. Parasitol..

[95] Sargison N. (2008). Sheep Flock Health—A Planned Approach.

[96] Verhoeven P.O., Gagnaire J., Botelho-Nevers E., Grattard F., Carricajo A., Lucht F., Pozzetto B., Berthelot P. (2014). Detection and clinical relevance of *Staphylococcus aureus* nasal carriage: An update. Expert Rev. Anti-Infect. Ther..

[97] Ganter M. (2015). Zoonotic risks from small ruminants. Vet. Microbiol..

[98] World Health Organization (2020). Zoonoses. https://www.who.int/news-room/fact-sheets/detail/zoonoses.

[99] Haydon D.T., Cleaveland S., Taylor L.H., Laurenson M.K. (2002). Identifying reservoirs of infection: A conceptual and practical challenge. Emerg. Inf. Dis..

[100] Blasco J.M., Molina-Flores B. (2011). Control and eradication of *Brucella melitensis* infection in sheep and goats. Vet. Clin. N. Am. Food Anim. Pract..

[101] Fthenakis G.C., Christodoulopoulos G., Leontides L., Tzora A. (2006). Abortion in ewes associated with *Erysipelothrix rhusiopathiae*. Small Rumin. Res..

[102] Cvetojevic D., Radanovic O., Milicevic V., Jezdimirovic N., Kureljusic B. (2017). Polyarthritis in goat-kids caused by *Streptococcus equi* subspecies *zooepidemicus*. Acta Vet. Beog..

[103] Wilson A.J., Morgan E.R., Booth M., Norman R., Perkins S.E., Hauffe H.C., Mideo N., Antonovics J., McCallum H., Fenton A. (2017). What is a vector?. Philos. Trans. R. Soc. Lond. B Biol. Sci..

[104] European Food Safety Authority (2026). Vector-Borne Diseases. https://www.efsa.europa.eu/en/topics/topic/vector-borne-diseases.

[105] de la Fuente J., Estrada-Pena A., Venzal J.M., Kocan K.M., Sonenshine D.E. (2008). Overview: Ticks as vectors of pathogens that cause disease in humans and animals. Front. Biosci..

[106] Stuen S. (2013). Tick-borne infections in small ruminants in northern Europe. Small Rumin. Res..

[107] Kampen H., Werner D. (2023). Biting midges (Diptera: Ceratopogonidae) as vectors of viruses. Microorganisms.

[108] Zehairy A.A., Sohrab S.S., Al-Judaibi A.A., Azhar E.I. (2026). Rift Valley Fever Virus: An update on current status and future prospects. Rev. Argent. Microbiol..

[109] Bergonier D., Berthelot X., Poumarat F. (1997). Contagious agalactia of small ruminants: Current knowledge concerning epidemiology, diagnosis and control. Rev. Sci. Tech. Off. Int. Epiz..

[110] Yin J.-H., Kelly P.J., Wang C. (2022). Flies as vectors and potential sentinels for bacterial pathogens and antimicrobial resistance: A review. Vet. Sci..

[111] Nichols E., Spector S., Louzada J., Larsen T., Amezquita S., Favila M.E. (2008). Ecological functions and ecosystem services provided by Scarabaeinae dung beetles. Biol. Conserv..

[112] Blanckenhorn W.U., Puniamoorthy N., Scheffczyk A., Römbke J. (2013). Evaluation of eco-toxicological effects of the parasiticide moxidectin in comparison to ivermectin in 11 species of dung flies. Ecotoxicol. Environ. Saf..

[113] Finch D., Schofield H., Floate K.D., Kubasiewicz L.M., Mathews F. (2020). Implications of endectocide residues on the survival of Aphodiine dung beetles: A meta-analysis. Environ. Toxicol. Chem..

[114] Tardiolo G., La Fauci D., Riggio V., Daghio M., Di Salvo E., Zumbo A., Sutera A.M. (2025). Gut microbiota of ruminants and monogastric livestock: An overview. Animals.

[115] Wang X.L., Zhou J., Lu M.L., Zhao S.P., Li W.J., Quan G.B., Xue B. (2025). Mucosal barrier function and microbial community of small intestines in sheep in response to dietary energy concentrations. Animal.

[116] Calvo-Bado L., Oakley B., Dowd S., Green L.E., Medley G.F., Ul-Hassan A., Bateman V., Gaze W., Witcomb L., Grogono-Thomas R. (2011). Ovine pedomics: The first study of the ovine foot 16S rRNA-based microbiome. ISME J..

[117] Cholewińska P., Nazar P., Junkuszew A., Smoliński J., Czyż K., Wyrostek A. (2021). The level of selected bacterial phyla on the skin surface of small ruminants according to the breed and species. Animals.

[118] Jost S.M., Cardona L., Rohrbach E., Mathis A., Holliger C., Verhulst N.O. (2024). Environment rather than breed or body site shapes the skin bacterial community of healthy sheep as revealed by metabarcoding. Vet. Dermatol..

[119] Fragkou I.A., Mavrogianni V.S., Cripps P.J., Gougoulis D.A., Fthenakis G.C. (2007). The bacterial flora in the teat duct of ewes can protect against and can cause mastitis. Vet. Res..

[120] Knuth R.M., Page C.M., Stewart W.C., Hummel G.L., Woodruff K.L., Whaley J.R., Springer A.L., Austin K.J., Murphy T.W., Bisha B. (2024). Milk microbiome in the first month of lactation and at weaning from ewes supplemented with zinc pre- and postpartum. J. Anim. Sci..

[121] Zhang Y., Bai X., Wang X., Lan L., Yang D., Zhang B., Diao E., Miao Y., Wu K., Wang B. (2025). Characteristics of the milk microbiota of healthy goats and goats diagnosed with clinical mastitis in Western China. Microb. Pathog..

[122] Qi W., Xue M.Y., Jia M.H., Zhang S., Yan Q., Sun H.Z. (2023). Understanding the functionality of the rumen microbiota: Searching for better opportunities for rumen microbial manipulation. Anim. Biosci..

[123] Yu Z., Yan M., Wang J. (2024). Rumen microbiome nutriomics: Harnessing omics technologies for enhanced understanding of rumen microbiome functions and ruminant nutrition. Anim. Nutr..

[124] Waters S.M., Roskam E., Smith P.E., Kenny D.A., Popova M., Eugène M., Morgavi D.P. (2025). The role of rumen microbiome in the development of methane mitigation strategies for ruminant livestock. J. Dairy Sci..

[125] Rodrigues A.A., Ferreira I.M., Umana F.V., de Oliveira J.S., Mantovani H.C. (2026). Rumen methanogenic archaea and their correlation with enteric methane emission in ruminant animals: A systematic review and meta-analysis. JSFA Rep..

[126] Okoye C.O., Wang Y., Gao L., Wu Y., Li X., Sun J., Jiang J. (2023). The performance of lactic acid bacteria in silage production: A review of modern biotechnology for silage improvement. Microbiol. Res..

[127] Acharya M., Ashworth A.J., Yang Y., Burke J.M., Lee J.A., Sharma Acharya R. (2021). Soil microbial diversity in organic and non-organic pasture systems. PeerJ.

[128] Wang Q., Chai Q., Dou X., Zhao C., Yin W., Li H., Wei J. (2024). Soil microorganisms in agricultural fields and agronomic regulation pathways. Agronomy.

[129] Songer J.G. (2010). Clostridia as agents of zoonotic disease. Vet. Microbiol..

[130] Uzal F.A., Navarro M.A., Li J., Freedman J.C., Shrestha A., McClane B.A. (2018). Comparative pathogenesis of enteric clostridial infections in humans and animals. Anaerobe.

[131] Golder H.M., Lean I.J. (2024). Ruminal acidosis and its definition—A critical review. J. Dairy Sci..

[132] Mao J., Wang L. (2025). Rumen acidosis in ruminants: A review of the effects of high-concentrate diets and the potential modulatory role of rumen foam. Front. Vet. Sci..

[133] Taylor D. (1999). Antimicrobial use in animals and its consequences for human health. Clin. Microbiol. Inf..

[134] Trinchera M., De Gaetano S., Sole E., Midiri A., Silvestro S., Mancuso G., Catalano T., Biondo C. (2025). Antimicrobials in livestock farming and resistance: Public health implications. Antibiotics.

[135] Ricklefs R.E. (2005). The Economy of Nature.

[136] Lianou D.T., Michael C.K., Fthenakis G.C. (2023). Data on mapping 444 dairy small ruminant farms during a countrywide investigation performed in Greece. Animals.

[137] Bandopadhyay S. (2016). Does elevation impact local level climate change? An analysis based on fifteen years of daily diurnal data and time series forecasts. Pac. Sci. Rev. A Nat. Sci. Eng..

[138] Antúnez P. (2023). Evidence of the variation in the rate of change of temperature and precipitation. Ecol. Informat..

[139] Katsarou E.I., Lianou D.T., Papadopoulos E., Fthenakis G.C. (2022). Long-term climatic changes in small ruminant farms in Greece and potential associations with animal health. Sustainability.

[140] Poux X. Low input farming systems in Europe: What is at stake?. Proceedings of the JRC XII International Symposium on Agricultural Sciences AgroReS.

[141] Gantner R., Steiner Z., Gantner V. (2023). Low-input farming for agricultural sustainability. AgroReS.

[142] Vasileiou N.G.C., Fthenakis G.C., Papadopoulos E. (2015). Dissemination of parasites by animal movements in small ruminant farms. Vet. Parasitol..

[143] Katsarou E.I., Lianou D.T., Michael C.K., Vasileiou N.G.C., Papadopoulos E., Petinaki E., Fthenakis G.C. (2024). Associations of climatic variables with health problems in dairy sheep farms in Greece. Climate.

[144] Taylor M.A., Coop R.L., Wall R.L. (2007). Veterinary Parasitology.

[145] Redding L., Huang E., Ryave J., Webb T., Barnhart D., Baker L., Bender J., Kristula M., Kelly D. (2021). *Clostridioides difficile* on dairy farms and potential risk to dairy farm workers. Anaerobe.

[146] Tarrah A., Zhang D., Darvishzadeh P., LaPointe G. (2024). The contribution of dairy bedding and silage to the dissemination of genes coding for antimicrobial resistance: A narrative review. Antibiotics.

[147] Michael C.K., Lianou D.T., Vasileiou N.G.C., Tsilipounidaki K., Katsafadou A.I., Politis A.P., Kordalis N.G., Ioannidi K.S., Gougoulis D.A., Trikalinou C. (2021). Association of staphylococcal populations on teatcups of milking parlours with vaccination against staphylococcal mastitis in sheep and goat farms. Pathogens.

[148] Arsenopoulos K.V., Diakidi D.S., Katsarou E.I., Michalopoulou E., Papadopoulos E., O’Doherty J., Vlasiou M., Fthenakis G.C. (2026). Water in livestock and poultry nutrition: A review on consumption and quality. Water.

[149] Talaga-Ćwiertnia K. (2021). *Sarcoptes* infestation. What is already known, and what is new about scabies at the beginning of the third decade of the 21st century?. Pathogens.

[150] McGrath T.T. (1929). On the presence of the Bacillus of Preisz-Nocard in infected shear-cut wounds in sheep. Aus. Vet. J..

[151] Serikawa S., Ito S., Hatta T., Kusakari N., Senna K., Sawara S., Hiramune T., Kikuchi N., Yanagawa R. (1993). Seroepidemiological evidence that shearing wounds are mainly responsible for *Corynebacterium pseudotuberculosis* infection in sheep. J. Vet. Med. Sci..

[152] Raadsma H.W., Egerton J.R. (2013). A review of footrot in sheep: Aetiology, risk factors and control methods. Liv. Sci..

[153] Corner L.A., Collins N.D., Vaughan J.A. (1996). An experimental ovine foot abscess model using a *Fusobacterium necrophorum* biotype AB. Vet. Microbiol..

[154] Egerton J.R., Martin W.B., Aitken I.D. (2000). Foot-rot and other foot conditions. Diseases of Sheep.

[155] Gaviglio A., Corradini A., Marescotti M.E., Demartini E., Filippini R. (2021). A theoretical framework to assess the impact of flooding on dairy cattle farms: Identification of direct damage from an animal welfare perspective. Animals.

[156] Zanolari P., Dürr S., Jores J., Steiner A., Kuhnert P. (2021). Ovine footrot: A review of current knowledge. Vet. J..

[157] Katsarou E.I., Lianou D.T., Michael C.K., Petridis I.G., Vasileiou N.G.C., Fthenakis G.C. (2024). Lameness in adult sheep and goats in Greece: Prevalence, predictors, treatment, importance for farmers. Animals.

[158] Broom D.M. (2019). Animal welfare complementing or conflicting with other sustainability issues. Appl. Anim. Behav. Sci..

[159] Steenaert L.I.W., Koop G. (2025). The effect of floor type on the welfare of intensively housed goats: A review. Small Rumin. Res..

[160] McIlroy S.G., Goodall E.A., McCracken R.M., Stewart D.A. (1989). Rain and windchill as factors in the occurrence of pneumonia in sheep. Vet. Rec..

[161] Lacasta D., Ferrer L.M., Ramos J.J., González J.M., De Las Heras M. (2008). Influence of climatic factors on the development of pneumonia in lambs. Small Rumin. Res..

[162] Liagka D.V., Fthenakis G.C., Mavrogianni V.S., Lianou D.T., Spyrou V., Vasileiou N.G.C. (2026). Machine milking in small ruminants: Milking systems and association with milk quality produced in the farms. Dairy.

[163] Liagka D.V., Fthenakis G.C., Kalonaki S.N., Dimoveli K.S., Lianou D.T., Mavrogianni V.S., Michael C.K., Caroprese M., Spyrou V., Vasileiou N.G.C. (2025). Machine-milking practices, animal welfare-related reactions and quality of milk produced in dairy sheep farms. Animals.

[164] Vasileiou N.G.C., Cripps P.J., Ioannidi K.S., Chatzopoulos D.C., Gougoulis D.A., Sarrou S., Orfanou D.C., Politis A.P., Calvo Gonzalez-Valerio T., Argyros S. (2018). Extensive countrywide field investigation of subclinical mastitis in sheep in Greece. J. Dairy Sci..

[165] Romero G., Peris C., Fthenakis G.C., Diaz J.R. (2020). Effects of machine milking on udder health in dairy ewes. Small Rumin. Res..

[166] Samelius G., Suryawanshi K., Frank J., Agvaantseren B., Baasandamba E., Mijiddorj T., Johansson O., Tumursukh L., Mishra C. (2021). Keeping predators out: Testing fences to reduce livestock depredation at night-time corrals. Oryx.

[167] Lima T.S., Sarmento Júnior F.D.C., Barbosa F.M.S., Melo E.T., Castillo S.M., Nascimento H.H.L., Kommers G.D., Lucena R.B. (2026). Traumatic Marjolin’s ulcer in sheep: Squamous cell carcinoma arising from chronic dorsal fence-wire injuries. Vet. J..

[168] Xu W., Dejid N., Herrmann V., Sawyer H., Middleton A.D. (2021). Barrier Behavior Analysis (BaBA) reveals extensive effects of fencing on wide-ranging ungulates. J. Appl. Ecol..

[169] Bennett D.E., Brammer T., Merkle J.A., Smith K.T., Regan S., Stoellinger T., Middleton A., Barker K., Hochard J.P., Lendrum P.E. (2026). Advancing conservation through virtual livestock fencing. Biol. Conserv..

[170] Hogan J., Phillips C.J.C. (2008). Nutrition and the welfare of ruminants. Ann. Rev. Biomed. Sci..

[171] González L.A., Kyriazakis I., Tedeschi L.O. (2018). Precision nutrition of ruminants: Approaches, challenges and potential gains. Animal.

[172] McGrath J., Duval S.M., Tamassia L.F.M., Kindermann M., Stemmler R.T., de Gouvea V.N., Acedo T.S., Immig I., Williams S.N., Celi P. (2018). Nutritional strategies in ruminants: A lifetime approach. Res. Vet. Sci..

[173] Bobeck E.A. (2020). Nutrition and health: Companion animal applications: Functional nutrition in livestock and companion animals to modulate the immune response. J. Anim. Sci..

[174] National Research Council (2007). Nutrient Requirements of Small Ruminants.

[175] Hooda P.S., Edwards A.C., Anderson H.A., Miller A. (2000). A review of water quality concerns in livestock farming areas. Sci. Tot. Environ..

[176] Manning P., van der Plas F., Soliveres S., Allan E., Maestre F.T., Mace G., Whittingham M.J., Fischer M. (2018). Redefining ecosystem multifunctionality. Nat. Ecol. Evol..

[177] McCracken N. (2026). Finding Dumped Animal Carcasses in Rivers Is Shameful’ Says Litter Picker. https://www.bbc.com/news/articles/crkpkm55yj4o.

[178] McManus C., Lima Dallago B.S., Lehugeur C., Ribeiro L.A., Hermuche P., Guimarães R.F., de Carvalho Junior O.A., Paiva S.R. (2016). Patterns of heat tolerance in different sheep breeds in Brazil. Small Rumin. Res..

[179] Marshall K., Gibson J.P., Mwai O., Mwacharo J.M., Haile A., Getachew T., Mrode R., Kemp S.J. (2019). Livestock genomics for developing countries–African examples in practice. Front. Genet..

[180] Singh M., More T., Rai A.K. (1980). Heat tolerance of different genetic groups of sheep exposed to elevated temperature conditions. J. Agric. Sci..

[181] Berihulay H., Abied A., He X., Jiang L., Ma Y. (2019). Adaptation mechanisms of small ruminants to environmental heat stress. Animals.

[182] Sawyer G., Narayan E.J., Narayan E. (2019). A review on the influence of climate change on sheep reproduction. Comparative Endocrinology of Animals.

[183] Ramón M., Carabaño M.J., Díaz C., Kapsona V.V., Banos G., Sánchez-Molano E. (2021). Breeding strategies for weather resilience in small ruminants in Atlantic and Mediterranean climates. Front. Genet..

[184] Serranito B., Cavalazzi M., Vidal P., Taurisson-Mouret D., Ciani E., Bal M., Rouvellac E., Servin B., Moreno-Romieux C., Tosser-Klopp G. (2021). Local adaptations of Mediterranean sheep and goats through an integrative approach. Sci. Rep..

[185] Hu Z., Wright I.J., Reich P.B., Knapp A.K., Ye G., Wang H., Chave J., Zeng X., Yang G., Dai L. (2026). Towards unifying precipitation-vegetation productivity relationships in terrestrial ecosystems. Fundam. Res..

[186] Liu Y., Li Z., Chen Y., Gao L., Zhang L., Turnadge C., He B., Zhou X., Duan W., Li B. (2026). Vegetation dynamics offset nearly 30% of precipitation’s impact on runoff and are amplified by aridity. One Earth.

[187] Tarpoff A., Smith Z., Brouk M. (2025). Mitigating impacts of environmental extremes in ruminant animals. Vet. Clin. Food Anim. Pract..

[188] Neher D.A., Andrews T.D., Weicht T.R., Hurd A., Barlow J.W. (2022). Organic farm bedded pack system microbiomes: A case study with comparisons to similar and different bedded packs. Dairy.

[189] Zhao X., Li J., Che Z., Xue L. (2022). Succession of the bacterial communities and functional characteristics in sheep manure composting. Biology.

[190] Li M., Jiang L., Li F., Zhang X., Zhao H., Duan N. (2025). Microbial agents assisted aerobic co-composting of sheep manure and furfural residue: Maturation enhancement and microbial dynamic. J. Environ. Chem. Engin..

[191] Fthenakis G.C. (2008). Veterinary specialization in small ruminant health management: Foundation of a European College of Small Ruminant Health Management [ECSRHM]. Small Rumin. Res..

[192] Wang H., McEntire J.C., Zhang L., Li X., Doyle M. (2012). The transfer of antibiotic resistance from food to humans: Facts, implications and future directions. Rev. Sci. Tech. Off. Int. Epiz..

[193] Schwarz S., Loeffler A., Kadlec K. (2017). Bacterial resistance to antimicrobial agents and its impact on veterinary and human medicine. Vet. Dermatol..

[194] Morgan-Davies C., Gautier J.M., González-García E., Halachmi I., Caja López G., Molle G., Kenyon F., Lagriffoul G., Schmoelzl S., Wishart H. Potential of Precision Livestock Farming in small ruminant farming systems. Proceedings of the 70th Annual Meeting of the European Federation of Animal Science.

[195] Caja G., Castro-Costa A., Salama A.A.K., Oliver J., Baratta M., Ferrer C., Knight C.H. (2020). Sensing solutions for improving the performance, health and wellbeing of small ruminants. J. Dairy Res..

[196] Morgan-Davies C., Tesnière G., Gautier J.M., Jørgensen G.H.M., González-García E., Patsios S.I., Sossidou E.N., Keady T.W.J., McClearn B., Kenyon F. (2024). Exploring the use of precision livestock farming for small ruminant welfare management. Animal.

[197] Fthenakis G.C. Use of machine learning in the health management of small ruminants. Proceedings of the 33rd Buiatrics Congress.

