# Supplementary material for: Small Ruminant Farms as Managed Ecosystems: A Conceptual Framework for Components and Interactions, and the Role of Health Management
Source: Animals (Basel). 2026 Sep 29;16(19):3067. doi: 10.3390/ani16193067 (PMC13663406; doi:10.3390/ani16193067)
Supplement: Supplementary file 1 [file animals-16-03067-s001.zip › animals-4556909-supplementary.pdf]

Small Ruminant Farms as Managed Ecosystems: A Conceptual Framework for Components and Interactions, and the Role of Health Management

E.I. Katsarou, D.C. Chatzopoulos, M.V. Bourganou, N.G.C. Vasileiou, I.A. Fragkou, V.S. Mavrogianni and G.C. Fthenakis

Table S1. Management systems applied in sheep farms and the principal elements of these systems (source: reproduced from the EFSA Panel on Animal Health and Welfare (AHAW) [1] under the Creative Commons Attribution–NoDerivatives licence [CC BY-ND]).

	Continuous presence of stockperson with sheep	No outdoor access	Housing [1]	Kept in fenced pastures [2]	Supplementation
Shepherding	Yes	[3]			
Intensive system	No	Yes			
Semi-intensive system	No	No	Yes		
Semi-extensive system	No	No	No	Yes	
Extensive system	No	No	No	No	Yes
Very extensive system	No	No	No	No	No

[1]: during the night and part of the day; [2]: including rotational grazing; [3]: blank cells in the table indicate that the criterion is not applicable to the respective systems.

Reference

1. EFSA AHAW Panel (EFSA Panel on Animal Health and Welfare). Scientific opinion on the welfare risks related to the farming of sheep for wool, meat and milk production. *EFSA J.* **2014**, *12*, 3933–4060.

Table S2. Examples of wildlife mammal predators for sheep or/and goats that predominate in various regions of the world.

Regions	Examples of predators	References
Africa	Black-backed jackals (*Lupulella mesomelas*), caracals (*Caracal caracal*), free-ranging domestic dogs (*Canis lupus familiaris*)	[2,3]
America	Coyotes (*Canis latrans*), free-ranging domestic dogs (*Canis lupus familiaris*), jaguars (*Panthera onca*), pumas (*Puma concolor*)	[4,5]
Asia	Free-ranging domestic dogs (*Canis lupus familiaris*), grey wolves (*Canis lupus lupus*), Himalayan wolves (*Canis lupus chanco*), snow leopards (*Panthera uncia*)	[6,7]
Europe	Brown bears (*Ursus arctos*), free-ranging domestic dogs (*Canis lupus familiaris*), golden jackals (*Canis aureus*), grey wolves	[8-10]
Oceania	Dingoes (*Canis lupus dingo*), free-ranging domestic dogs (*Canis lupus familiaris*)	[11]

Reference

2. Nattrass, N.; Conradie, B. Predators, livestock losses and poison in the South African Karoo. *J. Clean. Prod.* **2018**, *194*, 777-785.
3. Nattrass, N.; Drouilly, M.; O'Riain, M.J. Learning from science and history about black-backed jackals *Canis mesomelas* and their conflict with sheep farmers in South Africa. *Mamm. Rev.* **2020**, *50*, 101-111.
4. McInturff, A.; Miller, J.R.B.; Gaynor, K.M.; Brashares, J.S. Patterns of coyote predation on sheep in California: a socio-ecological approach to mapping risk of livestock-predator conflict. *Conserv. Sci. Pract.* **2021**, *3*, e175.
5. Torres-Romero, E.J.; Bender, L.C. Extent and patterns of livestock depredation in Mexico. *Rangel. Ecol. Manag.* **2024**, *96*, 23-31.
6. Chetri, M.; Odden, M.; Wegge, P. Snow leopard and Himalayan wolf: food habits and prey selection in the Central Himalayas, Nepal. *PLoS One* **2017**, *12*, e0170549.
7. Hamid, A.; Mahmood, T.; Fatima, H.; Hennelly, L.M. Origin, ecology and human conflict of gray wolf (*Canis lupus*) in Suleman Range, South Waziristan, Pakistan. *Mammalia* **2019**, *83*, 539-551.
8. Ballarín, J.; García-Serrano, A.; Herrero, J.; Reiné, R. Shepherds view of large carnivore recovery in the Pyrenees, Spain. *Animals* **2023**, *13*, 2088.
9. Brunschwig, G.; Pinot, A.; Brosse-Genevet, E.; Virapin, M.; Garde, L. Predation impacts of dogs on flocks of sheep in wolf-free areas: A synthesis based on surveys conducted in 11 breeding territories in France. *Small Rumin. Res.* **2026**, *260*, 107779.
10. Katsarou, E.I.; Reid, N.; Lianou, D.T.; Fthenakis G.C. Stress related to wild canid predators near dairy sheep farms associated with increased somatic cell counts in bulk-tank milk. *Sci. Rep.* **2024**, *14*, 3252.
11. Allen, L.R.; Sparkes, E.C. The effect of dingo control on sheep and beef cattle in Queensland. *J. Appl. Ecol.* **2001**, *38*, 76-87.

Table S3. Examples of pathogens (n = 162) that infect sheep or/and goats.

**(a) Prions**		
Scrapie prion *PrP*^{Sc} (misfolded protein)		
**(b) Viruses**		
Bluetongue virus	*Nairobivirus*	*Ovine papillomavirus* 1
Border disease virus	*Orf virus*	*Parainfluenza virus* type 3
Caprine arthritis encephalitis virus	*Orthobunyavirus akabaneense*	*Peste-des-petits-ruminants virus*
Crimean-Congo hemorrhagic fever virus	*Orthobunyavirus schmallenbergense*	*Progressive pneumonia virus*
Epizootic haemorrhagic disease virus	*Orthoflavivirus loupingi*	*Rift Valley fever phlebovirus*
Foot-and-mouth disease virus	*Ovine adenoviruses* A	*Rotavirus alphagastroenteritidis*
Goat pox virus	*Ovine adenoviruses* B	*Sheep pox virus*
Jaagsiekte sheep retrovirus	*Ovine adenoviruses* D	*Thogotovirus*
Lyssavirus rabies	*Ovine herpesvirus 2*	*Visna-maedi virus*
**(c) Bacteria**		
Actinobacillus seminis	*Dichelobacter nodosus*	*Mycoplasma ovipneumoniae*
Anaplasma phagocytophilum	*Ehrlichia ruminantium*	*Mycoplasma ovis*
Anaplasma ovis	*Erysipelothrix rhusiopathiae*	*Mycoplasma putrefaciens*
Bacillus anthracis	*Escherichia coli*	*Pasteurella multocida*
Bibersteinia trehalosi	*Fusobacterium necrophorum*	*Salmonella enterica* subsp. *enterica*
Brucella abortus	*Histophilus ovis*	serovar Abortusovis
Brucella melitensis	*Leptospira interrogans* complex and	*Salmonella enterica* subsp. *enterica*
Brucella ovis	other pathogenic *Leptospira* spp.	serovar Brandenburg
Campylobacter coli	*Listeria ivanovii*	*Salmonella enterica* subsp. *enterica*
Campylobacter fetus subsp. *fetus*	*Listeria monocytogenes*	serovar Typhimurium
Campylobacter jejuni	*Mammaliicoccus sciuri*	*Staphylococcus aureus*
Chlamydia abortus	*Mannheimia haemolytica*	*Staphylococcus caprae*
Clostridium chauvoei	*Moraxella ovis*	*Staphylococcus chromogenes*
Clostridium novyi	*Mycobacterium avium* subsp. *paratuberculosis*	*Staphylococcus epidermidis*
Clostridium perfringens type A	*Mycobacterium bovis*	*Staphylococcus simulans*
Clostridium perfringens type B	*Mycoplasma agalactiae*	*Staphylococcus xylosus*
Clostridium perfringens type C	*Mycoplasma arginini*	*Streptococcus dysgalactiae*
Clostridium perfringens type D	*Mycoplasma capricolum* subsp. *capricolum*	*Streptococcus ovis*
Clostridium tetani	*Mycoplasma capricolum*	*Streptococcus uberis*
Corynebacterium pseudotuberculosis	subsp. *capripneumoniae*	*Streptococcus equi* subsp. *zooepidemicus*
Coxiella burnetii	*Mycoplasma mycoides* subsp. *capri*	*Trueperella pyogenes*
Dermatophilus congolensis	*Mycoplasma conjunctivae*	
**(d) Fungi**		
Microsporum spp.	*Trichophyton verrucosum*	
**(e) Protozoa**		
Babesia motasi	*Eimeria ovinoidalis*	*Theileria lestoquardi*
Babesia ovis	*Giardia duodenalis*	*Theileria ovis*
Cryptosporidium parvum	*Neospora caninum*	*Toxoplasma gondii*
Eimeria arloingi	*Sarcocystis capracanis*	*Trypanosoma congolense*
Eimeria caprina	*Sarcocystis gigantica*	*Trypanosoma evansi*
Eimeria crandallis	*Sarcocystis hircicanis*	*Trypanosoma vivax*
Eimeria ninakohlyakimovae	*Sarcocystis tenella*	
**(f) Metazoan Endoparasites**		
Bunostomum trigonocephalum	*Lucilia* spp. larvae	*Strongyloides papillosus*
Calicophoron daubneyi	*Moniezia expansa*	*Taenia multiceps* metacestode
Calliphora spp. larvae	*Muellerius capillaris*	(*Coenurus cerebralis*)
Chabertia ovina	*Nematodirus battus*	*Taenia ovis* metacestode (*Cysticercus ovis*)
Cooperia curticei	*Nematodirus filicollis*	*Teladorsagia circumcincta*
Cystocaulus ocreatus	*Nematodirus spathiger*	*Thelazia californiensis*
Dicrocoelium dendriticum	*Neostrongylus linearis*	*Thelazia ovis*
Dictyocaulus filaria	*Oesophagostomum columbianum*	*Thelazia rhodesii*
Echinococcus granulosus sensu lato	*Oesophagostomum venulosum*	*Trichostrongylus axei*
hydatid cysts	*Oestrus ovis* larvae	*Trichostrongylus colubriformis*
Fasciola gigantica	*Paramphistomum cervi*	*Trichostrongylus vitrinus*

Fasciola hepatica	*Protostrongylus rufescens*	*Trichuris ovis*
Haemonchus contortus	*Schistosoma bovis*	*Wohlfahrtia* spp. larvae
Hypoderma spp. larvae		
	**(g) Ectoparasites**	
Amblyomma spp.	*Haemaphysalis punctata*	*Psorobia ovis*
Bovicola capri	*Hyalomma* spp.	*Psoroptes cuniculi*
Bovicola ovis	*Ixodes ricinus*	*Psoroptes ovis*
Chorioptes bovis	*Linognathus* spp.	*Rhipicephalus* spp.
Ctenocephalides felis	*Melophagus ovinus*	*Sarcoptes scabiei*
Dermacentor marginatus		
